# Interventions for management of post-stroke depression: A Bayesian network meta-analysis of 23 randomized controlled trials

**DOI:** 10.1038/s41598-017-16663-0

**Published:** 2017-11-28

**Authors:** Linghui Deng, Xuejun Sun, Shi Qiu, Yao Xiong, Yuxiao Li, Lu Wang, Qiang Wei, Deren Wang, Ming Liu

**Affiliations:** 1 Stroke Clinical Research Unit, Department of Neurology, West China Hospital, Sichuan University, Chengdu, Sichuan China; 2 Second Department of Psychiatry, Kangning Hospital, Anshan, Liaoning China; 3 Department of Urology, Institute of Urology, West China Hospital, Sichuan University, Chengdu, Sichuan China

## Abstract

Post-stroke depression (PSD) is an important complication of stroke, leading to increased disability and mortality. Given that there is no consensus on which treatment is optimal for PSD, we aimed to evaluate the relative efficacies of available pharmacological and non-pharmacological interventions. We conducted a network meta-analysis to incorporate evidence from relevant trials and provide direct and indirect comparisons. We searched PubMed, Cochrane Library Central Register of Controlled Trials, and Embase until November 1, 2016 for randomized controlled trials involving different pharmacological and non-pharmacological PSD treatment interventions. The primary outcome was reduction in the Hamilton depression scale (HAMD) score. This study is registered with PROSPERO (number, CRD42016049049). Of a total of 1,152 studies, 23 randomized trials comprising 1,542 participants were included. Nine PSD treatment interventions were considered. Noradrenaline reuptake inhibitor (NRI) was associated with the highest reduction in the HAMD score, followed by tricyclic antidepressant (TCA), psychotherapy plus antidepressant, and selective serotonin reuptake inhibitor (SSRI). This study indicated that NRIs, SSRIs, and TCAs are associated with a considerable higher HAMD score reduction compared with the control treatment. rTMS is a beneficial therapeutic approach for managing PSD to obtain good response to treatments compared with the control treatment.

## Introduction

Globally, stroke is one of the leading causes of death and disability, and depression is a common sequela of stroke. Post-stroke depression (PSD) occurs in 31% of stroke survivors according to a recent meta-analysis of 61 cohort studies^1^, causing great burden to patients and their families. Several studies have suggested that PSD is associated with reduced quality of life and increased natural and suicidal deaths^2–5^.

The diagnosis of PSD can be complicated because of overlapping of some physical symptoms, such as cognitive and language impairments, associated with stroke. Moreover, various screening tools and diagnostic standards contribute to the challenge of identifying PSD. Consequently, only a small fraction of patients are accurately diagnosed and receive relevant treatment^5,6^.

The abrupt nature of stroke, resultant depression, and disability convolute the relationship between stroke and PSD. The pathogenesis of PSD remains controversial with respect to whether PSD is a direct consequence of specific neuroanatomical impairment or an indirect result of a patient’s negative psychological response to a stroke-related impairment^7^. Many factors such as stroke severity, lesion location, and functional and cognitive impairment may contribute to PSD development^8^. Studies have demonstrated that the incidence of depression was significantly higher in stroke survivors compared with that in a reference population without stroke^2^ but with comparable physical impairments^9^. Moreover, PSD was more likely distinguished from other types of late-life depression by a sad facial expression, depressive ideation, and vegetative symptoms^10^. In addition, studies suggested that depression severity was an independent predictive factor of the severity of an impairment among stroke survivors in performing daily activities and that depression has detrimental effects on rehabilitation and functional recovery after stroke^5,11^. Given that PSD differs from other types of depression in potentially unique ways, simply extrapolating data of treatment approaches for population with general depression to patients with PSD may be inappropriate.

Several therapeutic strategies for PSD have proved to be effective, including pharmacological and non-pharmacological interventions [e.g., psychotherapy and electroconvulsive therapy (ECT)]. Antidepressants are the most studied strategies, whereas the best characterized agents are fluoxetine, sertraline, citalopram, and nortriptyline^12^. The main goals of PSD treatments include reduction of depressive symptoms and complete remission (no longer meeting the baseline criteria for depression)^13^. Meta-analyses found antidepressants to be significantly effective in reducing depressive symptoms^13,14^. However, when assessed by Diagnostic and Statistical Manual of Mental Disorders (DSM) or Hamilton Depression Rating Scale (HAMD), no clear evidence was found on whether antidepressants are effective for complete remission of PSD^13,15^. Furthermore, Hackett *et al*. found no superiority over control intervention for psychotherapy alone^13^. Although selective serotonin reuptake inhibitors (SSRIs) are gaining popularity as first-line treatment for PSD and late-life depression^12,16^, neither studies provide conclusive evidence with respect to the superiority of SSRIs over any other treatments nor strong data recommend one particular SSRI over another for PSD management.

Despite the numerous therapeutic interventions, including both pharmacological and non-pharmacological interventions, evaluated in previous randomized controlled trials (RCTs) to treat PSD, majority have not been quantitatively analyzed in head-to-head comparisons. Thus, we performed a network meta-analysis (NMA) of all RCTs involving PSD treatment approaches, including pharmacological, non-pharmacological, and combination therapies, to comprehensively rank all available PSD treatments.

## Results

### Search and selection

**Figure 1:**
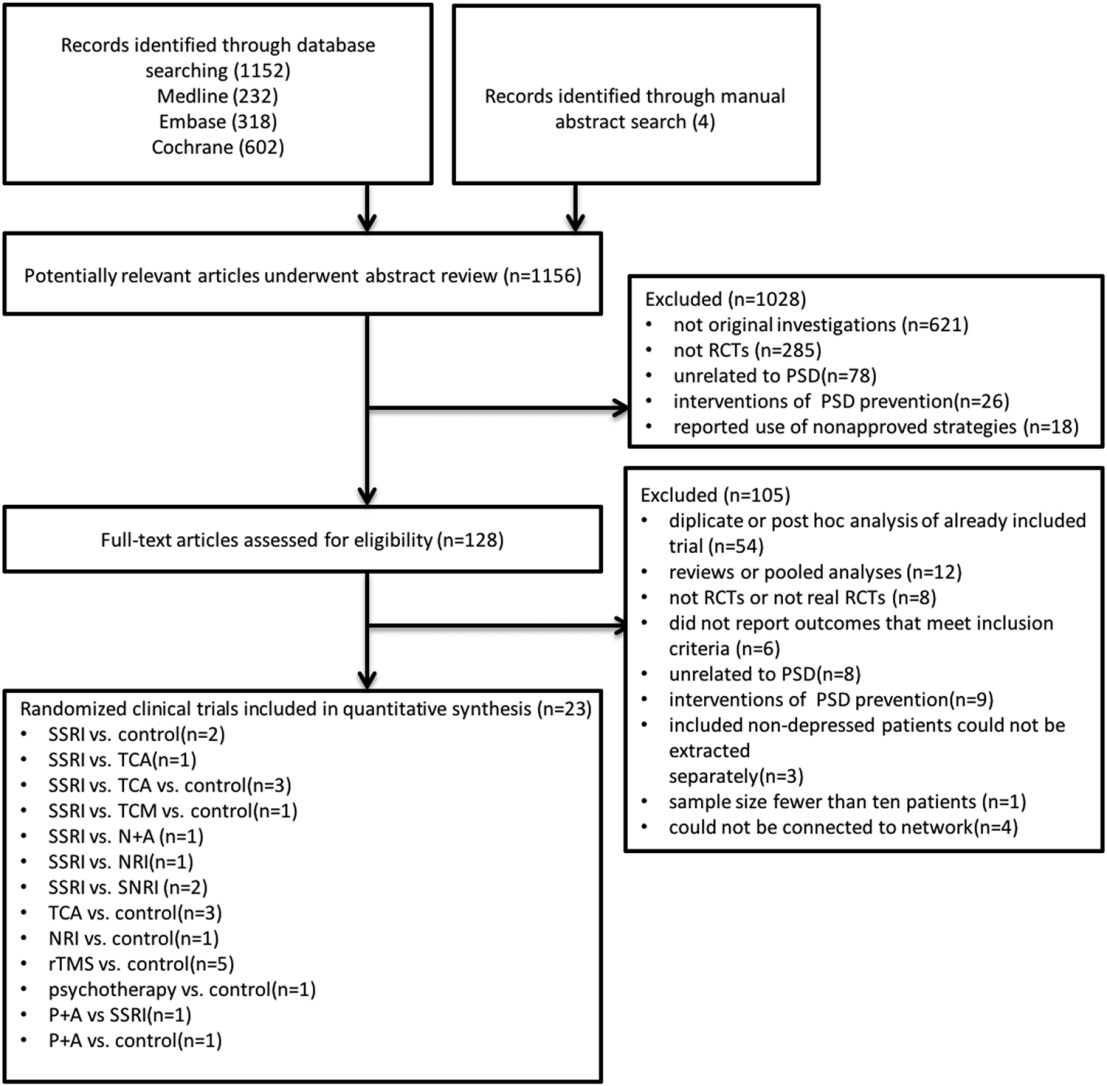
PRISMA flow diagram. SSRI = selective serotonin reuptake inhibitor. TCA = tricyclic antidepressant. SNRI = serotonin–norepinephrine reuptake inhibitors. NRI = norepinephrine reuptake inhibitor. TCM = traditional Chinese medicine. rTMS = Repetitive Transcranial Magnetic Stimulation. P + A = psychotherapy plus antidepressants. N + A = nimodipine plus antidepressants. RCT = randomized controlled trial.

From 1,152 records identified using the search algorithm, 23 RCTs, including 1,542 participants, were included in this NMA (Appendix 3). The systematic reviews and meta-analyses (PRISMA) flowchart depicting electronic searching processes is presented in Fig. 1.

### Characteristics of studies and participants

**Figure 2:**
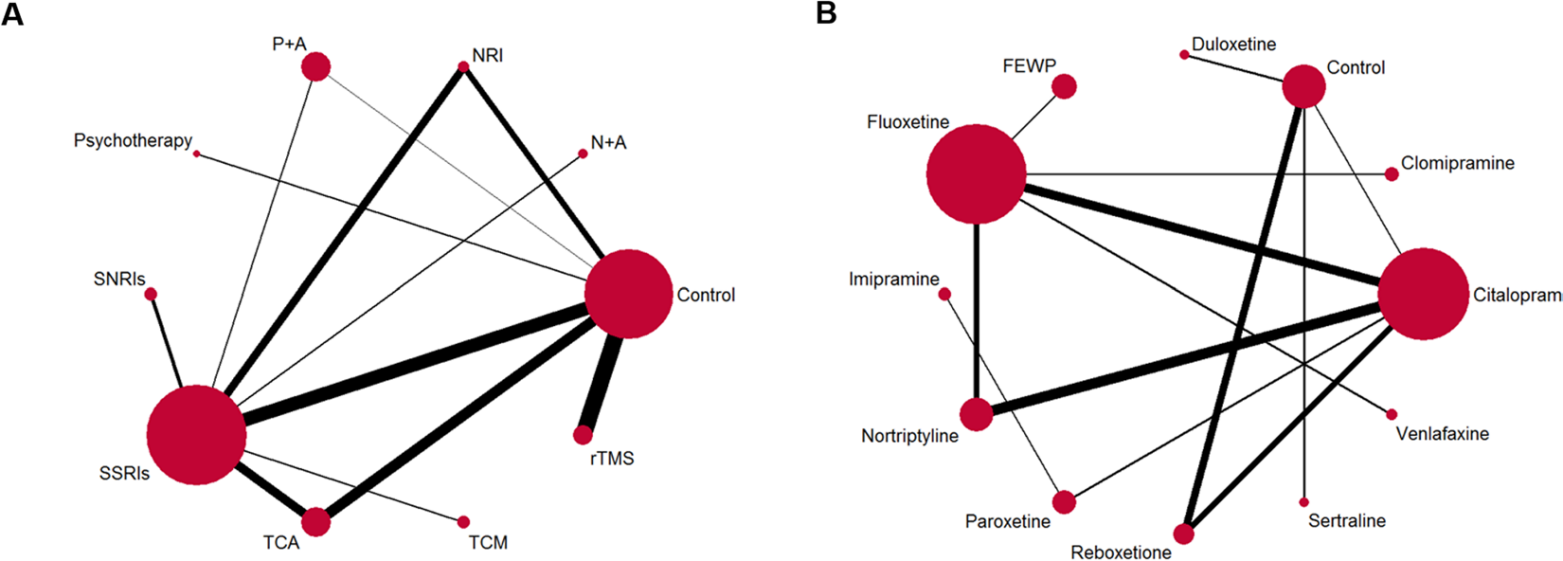
Network diagram of eligible comparisons. (**A**) Network diagram of eligible comparisons for reduction of HAMD score between individual treatment. (**B**) Network diagram of eligible comparisons for reduction of HAMD score between individual pharmacotherapy. The width of each line is proportional to the number of trials comparing every pair of treatments, and the size of each circle is proportional to the number of randomly allocated participants (sample size). SSRI = selective serotonin reuptake inhibitor. TCA = tricyclic antidepressant. SNRI = serotonin–norepinephrine reuptake inhibitors. NRI = norepinephrine reuptake inhibitor. TCM = traditional Chinese medicine. rTMS = Repetitive Transcranial Magnetic Stimulation. P + A = psychotherapy plus antidepressants. N + A = nimodipine plus antidepressants. FEWP = Free and Easy Wanderer Plus (a kind of Chinese medicine; its original Chinese name is Jia-Wei-Xiao-Yao-San).

The trials were published between February 1984 and October 2016, comparing nine different interventions. The number of patients allocated to each group ranged from 11 to 93. A total of 19 trials were two arm and four were three arm. SSRIs and the control are the two most frequent comparators across the studies. Figure 2 and Appendix 6 shows the available direct comparisons and network of trials. For the primary outcome, 11 of 45 pairwise comparisons had direct evidence. Detailed study characteristics are provided in Table 1. Moreover, 16 (69.6%) studies employed DSM as depression diagnostic criteria. The settings for recruited patients were inpatient (67.0%), outpatient (12.0%), and mixed (21.0%). Three trials only recruited patients diagnosed with major depression, nine trials included patients with both major and minor depression, and the remaining 11 trials did not clearly specify this aspect. The treatment duration ranged from 2 weeks to 6 months. The time of follow-up ranged from 2 weeks to 24 months. Studies were mostly multicenter site studies (56.5%). Table 2 summarizes the patient characteristics of NMA. Across trials, patient mean age ranged from 57 to 77.5 years, and approximately 48.8% of participants were male. The mean baseline HAMD score ranged from 10 to 32. A more detailed description of studies and treatments is provided in Appendix 4.

**Table 1:** Study characteristic.

Study	Location	Participants (N)	Intervention/control (N)	Drop-out rate (%)	Treatment duration	Follow-up	Setting	Center	Depression Diagnostic criteria	Population
Lipsey^33^	US	39	Nortriptyline 17	35.3	6 weeks	6 weeks	mixed	multi-center	DSM III	PP
			Placebo 22	31.8						
Andersen 1994	Denmark	66	Citalopram 33	21.2	6 weeks	16 weeks	mixed	multi-center	DSM III	ITT, PP
			Placebo 33	6.06						
Gonzalez 1995	Belgium	48	Fluoxetine 26	3.8	6 weeks	6 weeks	inpatient	single center	RDC	PP
			Nortriptyline 11	9.1						
			Control 11	9.1						
Robinson 2000	US	56	Fluoxetine 23	39.1	12 weeks	12 weeks	inpatient	multi-center	DSM-IV	ITT,PP
			Nortriptyline 16	18.8						
			Placebo 17	23.5						
Kimura 2000	US	47	Nortriptyline 21	14.3	6 or 12 weeks	12 weeks	inpatient	multi-center	DSM-IV	PP
			Placebo 26	0						
Taragano 2001	US	84	Nimodipine + SSRI 40	8.33 in total	60 d	300 day	inpatient	multi-center	DSM-IV	ITT
			SSRI 44							
Fruehwald 2003	Austria	54	Fluoxetine 28	7.14	12 weeks	18 months	inpatient	multi-center	NR	PP
			Placebo 26	7.7						
Kimura 2003	US	27	Nortrityline 13	7.7	6 or 12 weeks	12 weeks	inpatient	multi-center	DSM-IV	ITT
			Placebo 14	0						
Rampello^21^	Italy	74	Citalopram 37	8.1	16 weeks	16 weeks	outpatient	community-based	DSM-IV	PP
			Reboxetine 37	8.1						
Rampello^22^	Italy	31	Reboxetine 16	0	16 weeks	16 weeks	outpatient	community-based	DSM-IV	ITT
			Placebo 15							
Huang 2005	China	60	Fluoxetine 30	0	12 weeks	12 weeks	inpatient	single center	CCMD	ITT
			Clomipramine 30							
Ye 2006	China	90	Paroxetine 30	3.3	12 weeks	12 weeks	inpatient	single center	NR	PP
			Imipramine 30	1						
			Control 30	1						
Li 2008	China	150	TCM 60	0	8 weeks	8 weeks	inpatient	single center	NR	ITT
			Fluoxetine 60	3.3						
			Placebo 30	6.7						
Cravello 2009	Italy	50	Fluoxetine 25	0	8 weeks	8 weeks	inpatient	single center	DSM-IV	ITT
			Venlafaxine 25							
Dimitrios 2012	Greece	60	Duloxetine 20	0	3 months	3 months	outpatient	single center	DSM-IV	ITT
			Citalopram 20							
			Sertraline 20							
Jorge^23^	US	20	Active rTMS 10	0	2 weeks	3 weeks	outpatient	multi-center	DSM-IV	ITT
			Sham rTMS 10							
Jorge^24^	US	92	Active rTMS 48	0	3 weeks	3 weeks	mixed	multi-center	DSM-IV	ITT
			Sham rTMS 44							
Narushima^25^	US	65	Active rTMS 43	20.1	2 weeks	2 weeks	mixed	multi-center	DSM-IV-TR	PP
			Sham rTMS 22	50						
Tenev 2010	US	62	Active rTMS 33	0	2 weeks	3 weeks	mixed	multi-center	DSM-IV-TR	ITT
			Sham rTMS 29							
Seo 2016	Korea	24	Active rTMS 12	0	2 weeks	6 weeks	inpatient	single center	NR	ITT
			Sham rTMS 12							
Feng 2004	China	60	Psychotherapy 30	0	6 months	6 months	inpatient	single center	CES-D	ITT
			Control 30							
Williams 2007	US	182	P + A 89	5.6	12 weeks	12 weeks	inpatient	multi-center	DSM-IV	ITT
			Control 93	6.5						
Mitchell 2009	US	101	P + A 48	8.3	8 weeks	24 months	inpatient	multi-center	DSM-IV	PP
			SSRI 53	9.4						

DSM = Diagnostic and Statistical Manual of Mental Disorders.

DSM-IV-TR = Diagnostic and Statistical Manual of Mental Disorders fourth edition, text revision.

RDC = Research Diagnostic Criteria.

CCMD = Chinese Classification of Mental Disorder.

TCM = traditional Chinese medicine.

CES-D = center of epidemiological survey depression scale.

SSRI = selective serotonin reuptake inhibitor.

rTMS = Repetitive Transcranial Magnetic Stimulation.

P + A = Psychotherapy plus antidepressants therapy.

NR = not reported.

**Table 2:** Patient characteristics.

Study	intervention/control (N)	Mean age (SD)	Sex (%, male)	Mean baseline HAMD (SD)	Hemisphere stroke side (%, left)	Depression diagnosis N (%, major depression)	Time since stroke onset
Lipsey^33^	Nortriptyline(N = 14)	62(9)	64	13.9(0.79)	50	7(50%)	262(437) days
	Placebo(N = 20)	60(12)	65	16.57(0.85)	34	12(60%)	128(190) days
Andersen 1994	Citalopram(N = 33)	68.2(4.2)	36	19.4(3.1)	36.4	NR	10.6(9.8) weeks
	Placebo(N = 33)	65.8(9.0)	42	18.9(2.8)	39.4		13.2(11.0) weeks
Gonzalez 1995	Fluoxetine(N = 26)	66.71(12.60)^a^	52	23.52	47.9	34(71%)	within 4 weeks
	Nortriptyline(N = 11)			21.48			
	Control(N = 11)			23.52			
Robinson 2000	Fluoxetine(N = 23)	65(14)	74	20.4(4.7)	39.1	11(48%)	within 6 months
	Nortriptyline(N = 16)	64(10)	31	22.5(8.5)	37.5	10(63%)	
	Placebo(N = 17)	73(8)	53	17.5(6.2)	29.4	6(35%)	
Kimura 2000	Nortrityline(N = 21)	59.6(9.1)	47.6	17.38(4.3)	57.1	14(67%)	111(137) days
	Placebo(N = 26)	60.7(11.8)	65.4	17.92(3.95)	42.3	19(73%)	190(243) days
Taragano 2001	Nimodipine + SSRI(N = 40)	69.1(8.7)	33	26.8(5.8)	NR	100%	NR
	SSRI(N = 44)	68.4(7.1)	25	25.5(4.4)			
Fruehwald 2003	Fluoxetine(N = 28);	64.8(13.8)	46.2	32.8(12.7)	30.8	NR	11.0(3.9) days
	Placebo(N = 26)	64.0(14.3)	70.8	30.3(15.0)	50		11.1 ± 3.5 days
Kimura 2003	Nortrityline(N = 13)	64.8(11.3)	46.2	17.0(4.8)	46.2	6(46%)	73(101) days
	Placebo(N = 14)	55(15.2)	50.0	17.4(4.0)	35.7	11(79%)	117(159) days
Rampello^21^	Citalopram(N = 37)	73.13(4)	45.9	22.54(1.87)	48.6	NR	13.64(5.33) weeks
	Reboxetine(N = 37)	74.71(4.66)	48.6	22.76(2.02)	40.5		12.66(4.47) weeks
Rampello^22^	Reboxetine(N = 16);	77.5(4)	43.8	24.06(1.52)	56.3	NR	12.06(4.23) weeks
	Placebo(N = 15)	77.26(3.6)	46.7	24 (1.31)	56.3		12.26(4.77) weeks
Huang 2005	Fluoxetine(N = 30)	58(6)	56.7	21.3(2.64)	NR	NR	NR
	Clomipramine(N = 30)	NR	NR	20.09(2.1)			
Ye 2006	Paroxetine(N = 30)	58.06(8.46)	73.3	25.18(7.02)	60	NR	NR
	Imipramine(N = 30)	56.98(11.42)	60.0	24.2(9.04)	60		
	Control(N = 30)	59.37(9.56)	56.7	25.12(5.19)	63.3		
Lian 2008	TCM (N = 60)	68.5(4.10)	46.7	25.2(3.8)	58.3	NR	within 6 weeks
	Fluoxetine(N = 60)	69.2(3.5)	41.7	25.5(3.1)	51.7		
	Placebo(N = 30)	67.8(3.90)	56.7	24.3(2.90)	40		
Cravello 2009	Fluoxetine(N = 25)	65.9(12.7)	36	19.2(4.4)	NR	100%	146.8(41.5) days
	Venlafaxine(N = 25)	64.2(14.1)	44	17(4.5)			147.6(47.9) days
Dimitrios 2012	Duloxetine(N = 20)	51.1 (13.4)	NR	24.5 (7.5)	NR	NR	within 12 months
	Citalopram(N = 20)	54.3 (12.5)		23.7 (6.7)			
	Sertraline(N = 20)	52.4 (11.4)		23.8 (7.3)			
Jorge^23^	Active rTMS(N = 10)	63.1(8.1)	60	20.1(6.7)	NR	8(80%)	17.8(14.3) months
	Sham rTMS(N = 10)	66.5(12.2)	50	20.8(6.0)		9(90%)	
Jorge-12K^24^ ^d^	Active rTMS(N = 15)	62.9(7.2)	60	19.5(5.8)	NR	12(80%)	NR
	Sham rTMS(N = 15)	66.1(11.0)	47	19.9(5.4)		12(80%)	
Jorge-18K^24^ ^d^	Active rTMS(N = 33)	64.3(9.4)	39	18.4(3.4)	NR	28(85%)	NR
	Sham rTMS(N = 29)	62.1(8.5)	41	17.6(5.6)		22(76%)	
Narushima^25^	Active rTMS(N = 32)	61.5(2.5)^b^	40.6	16.52(1.6)	NR	100%	NR
	Sham rTMS(N = 11)		45.5	16.8(1.9)			
Tenev 2010	Active rTMS(N = 33)	64.5(8.9)	39	18.7(2.9)	NR	28(85%)^c^	NR
	Sham rTMS(N = 29)	63.3(8.5)	41	17.6(4.6)		22(77%)	
Seo 2016	Active rTMS(N = 12)	58.1(8.7)	50.0	10.0(1.3)	NR	NR	10.3(2.7) months
	Sham rTMS(N = 12)	58.3(7.8)	41.7	10.0(0.9)			10.1(2.3) months
Feng 2004	Psychotherapy(N = 30)	67.21(10.12)	60.0	12.1(3.4)	53.3	NR	NR
	Control(N = 30)	66.38(9.07)	53.3	13.7(3.8)	56.7		
Williams 2006	P + A(N = 89)	60(13)	39	18.0(5.4)	NR	64(%)	within 2 months
	Control(N = 93)	60(11)	52	19.2(5.9)		70(%)	
Mitchell 2009	P + A(n = 48)	57(25–88)	60.4	20.0(4.53)	37.5	NR	within 4 months
	SSRI(N = 53)	57(29–88)	60.4	19.8(4.15)	52.8		

^a^Pooled data from 2 groups: major depression group 67(13); minor depression group 66(12).

^b^Pooled data from 2 groups: responder group 60.1(2.2); non-responder group 65.9(2.0).

^c^All patients had major depression during the current depressive episode, but some were partially treated and met only DSM-IV-TR minor depression criteria when enrolled in the study.

^d^The trial divided patients into 2 group according to the total cumulative dose(TCD) the active groups accepted.

TCM = traditional Chinese medicine.

SSRI = selective serotonin reuptake inhibitor.

rTMS = Repetitive Transcranial Magnetic Stimulation.

P + A = Psychotherapy plus antidepressants therapy.

NR = not reported.

### Quality assessment and quality of the evidence

The risk of bias was high or unclear for random sequence generation in 14 trials; concealment of treatment allocation in 14 trials; masking of participants, masking of investigators, or both in eight trials; completeness of outcome reporting in three trials; and selective reporting of outcomes in three trials. None of the studies accepted financial funding from commercial bodies, and source of funding was unclear in 10 trials. We did not find any evidence of small study effects based on funnel plot asymmetry except for HAMD score change, although the number of studies recruited in each comparison was relatively small (Appendix 5). According to the grading of recommendations, assessment, developmental and evaluations (GRADE), most of the trials (7/9) were of moderate evidence quality (Table 3).

**Table 3:** Comparing evidence from the Network meta-analysis with evidence obtained from the pairwise meta-analysis.

Comparisons	Pairwise meta-analysis odds ratios (95% CI)	Network meta-analysis odds ratios (95% CrI)	No. of participants	No. of trials	No. of events	P-value	Heterogeneity I^2^	Quality of evidence	Downgraded reason
**HAMD score change for different treatment**									
rTMS vs. Control^1^	1.43 (1.06 to 1.79)	3.57 (−0.62 to 7.52)	179	5	—	0.01	89.7%	⊕⊕OO low	inconsistency and imprecision
SSRI vs. SNRI^2^	0.12 (−0.27 to 0.51)	0.58 (−6.13 to 6.98)	110	2	—	0.55	51.5%	⊕⊕OO low	heterogeneity and imprecision
SSRI vs. TCA^3^	0.07 (−0.24 to 0.38)	−1.32 (−5.45 to 2.56)	178	4	—	0.67	81.9%	⊕⊕⊕O moderate	heterogeneity
SSRI vs. NRI^4^	0.47 (−0.35 to 1.28)	−1.63 (−7.24 to 3.89)	68	2	—	0.26	98.8%	⊕⊕⊕O moderate	heterogeneity
TCA vs. Control^5^	1.29 (0.74 to 1.68)	7.64 (3.89 to 11.07)	97	3	—	0.01	91.8%	⊕⊕⊕O moderate	heterogeneity
SSRI vs. Control^6^	1.03 (0.78 to 1.28)	6.27 (2.66 to 9.69)	320	6	—	0.01	90.7%	⊕⊕⊕O moderate	heterogeneity
**Response rate for different treatment**									
SSRI vs TCA^7^	0.78 (0.47 to 1.30)	0.44 (0.23 to 0.83)	155	3	100	0.34	60.6%	⊕⊕⊕O moderate	heterogeneity
SSRI vs Control^8^	1.63 (1.02 to 2.67)	3.55 (1.98 to 6.46)	232	4	113	0.05	55.8%	⊕⊕⊕O moderate	heterogeneity
rTMS vs Control^9^	5.26 (2.17 to 12.5)	9.98 (4.06 to 27.96)	113	1	56	0%	0%	⊕⊕⊕O moderate	imprecision
**Remission rate for different treatment**									
SSRI vs Control^10^	2.38 (1.04 to 5.45)	3.24 (1.30 to 8.79)	115	2	34	0.04	0%	⊕⊕⊕O moderate	imprecision
rTMS vs Control^11^	4.72 (1.29 to 17.24)	2.25 (1.17 to 4.54)	155	1	25	0.01	0%	⊕⊕⊕O moderate	imprecision

All mean difference or odds ratios in bold are statistically significant. 95% CI = 95% Confidence Intervals. 95% CrI = 95% Credible Intervals. Using GRADE to rate quality of evidence from a network meta-analysis involved several steps: The pairwise meta-analyses (DerSimonian and Laird random effects model) of these two comparisons were conducted and are reported here in comparison with the estimates from the network analysis. The table shows comparison of estimates from pairwise meta-analysis compared to NMA. Quality of evidence as judged based on the Grading of Recommendations Assessment, Development and Evaluation (GRADE) approach. First, we rated quality of evidence for direct comparisons; second, we rated quality of evidence for indirect estimates (starting at the lowest rating of the two pairwise direct estimates that contribute as first-order loops to the indirect estimate, which can be rated down further for imprecision or intransitivity), and then third, rating the quality of evidence for the network combining direct and indirect estimates. In this step, if direct and indirect estimates from second-order comparisons are similar, the higher of the ratings was assigned to the network meta-analysis estimates.

SSRI = selective serotonin reuptake inhibitor.

TCA = tricyclic antidepressant.

SNRI = serotonin–norepinephrine reuptake inhibitors.

NRI = norepinephrine reuptake inhibitor.

rTMS = Repetitive Transcranial Magnetic Stimulation.

### Network consistency

The networks of individual intervention endpoints are presented in the appendix. There was no inconsistency in NMA estimates when we used the node-splitting approach and no significant differences between direct and indirect estimates in closed loops that allowed assessment of network coherence (Appendix 7). The total residual deviance for overall change in the HAMD score (45.9, df = 45), response rate (22.8, df = 21), and remission rate (15.6, df = 15) implied a good model fit. Convergence of chains was verified visually by looking at trace plots and inspecting the Brooks–Gelman–Rubin diagnostic statistics with values around 1.

### Pairwise and network result

For the primary outcome, active repetitive transcranial magnetic stimulation (rTMS), tricyclic antidepressants (TCAs), and SSRIs were significantly better than the control treatment [mean difference (MD) 1.43, 95% confidence interval (CI) 1.06–1.79; MD 1.29, 95% CI 0.74–1.68; MD 1.03, 95% CI 0.78–1.28]. For response and remission rates, rTMS was profoundly more effective than the control treatment [odds ratio (OR) 5.26, 95% CI 2.17–12.5; OR 4.72, 95% CI 1.29–17.24]. SSRIs were also better than the control treatment (OR 1.63, 95% CI 1.02–2.67; OR 2.38, 95% CI 1.04–5.45) (Table 3).

**Figure 3:**
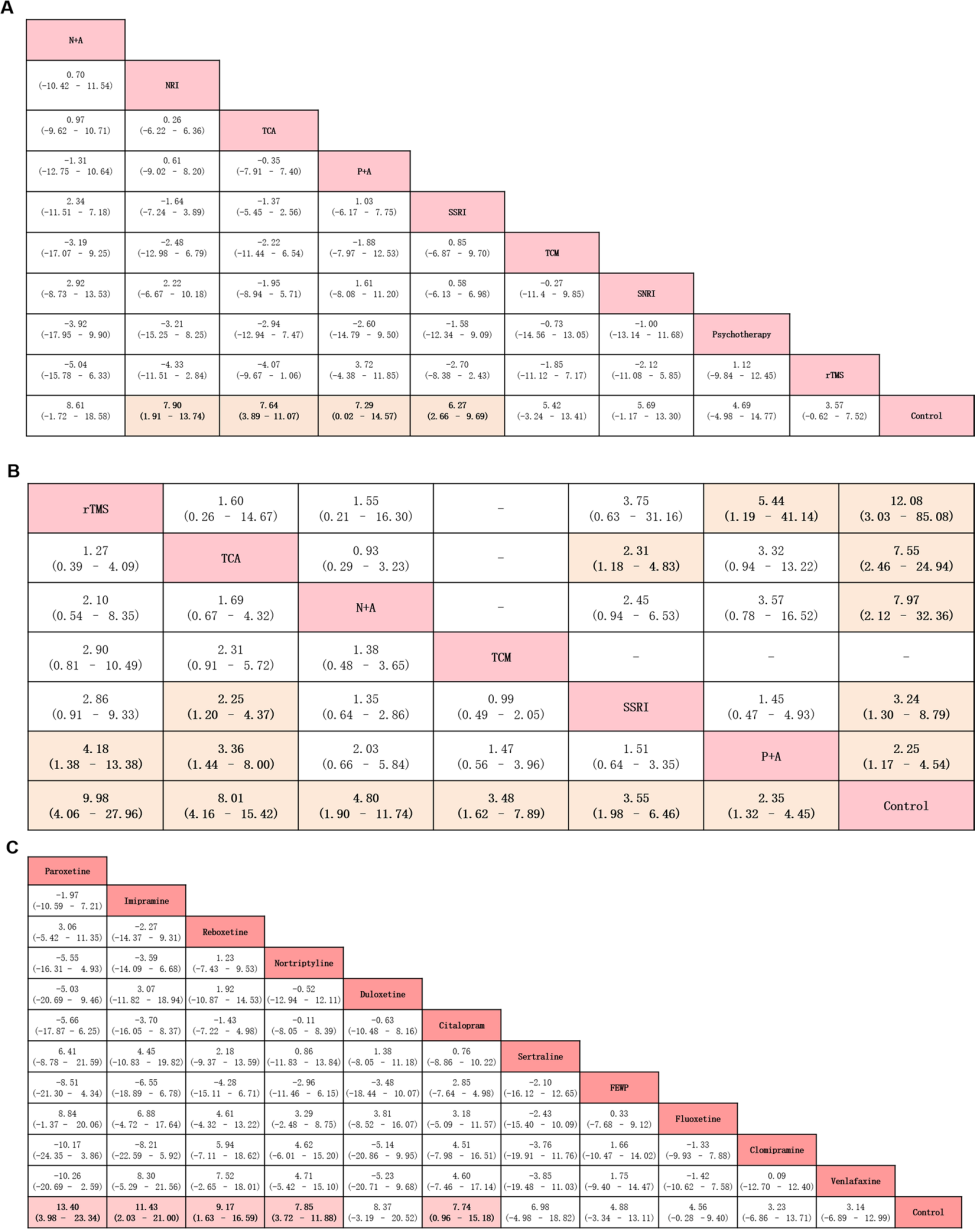
Summary results of network meta-analysis. (**A**) Summary mean difference and credible intervals from network meta-analysis of HAMD score change of individual treatment. Treatments are reported in order of efficacy ranking according to SUCRAs. Comparisons should be read from left to right. The efficacy estimate is located at the intersection of the column-defining treatment and the row-defining treatment. For efficacy (mean overall change in symptoms), an MD below 0 favours the column-defining treatment. To obtain MDs for comparisons in the opposing direction, negative values should be converted into positive values and vice versa. Significant results are in bold and underlined. SSRI = selective serotonin reuptake inhibitor. TCA = tricyclic antidepressant. SNRI = serotonin–norepinephrine reuptake inhibitors. NRI = norepinephrine reuptake inhibitor. TCM = traditional Chinese medicine. rTMS = Repetitive Transcranial Magnetic Stimulation. P + A = psychotherapy plus antidepressants. N + A = nimodipine plus antidepressants. (**B**) Summary odds ratio and credible intervals from network meta-analysis of response rate and remission rate of individual treatment. Treatments are reported in order of efficacy ranking according to SUCRAs. Comparisons should be read from left to right. The response rate (lower left portion) and remission rate (upper right portion) meta-analytic results are shown for the primary outcome. The response rate and remission rate estimate is located at the intersection of the column-defining treatment and the row-defining treatment. An OR value below 1 favours the column-defining treatment. To obtain ORs for comparisons in the opposing direction, reciprocals should be taken. Significant results are in bold and underlined. SSRI = selective serotonin reuptake inhibitor. TCA = tricyclic antidepressant. TCM = traditional Chinese medicine. rTMS = Repetitive Transcranial Magnetic Stimulation. P + A = psychotherapy plus antidepressants. N + A = nimodipine plus antidepressants. (**C**) Summary mean difference and credible intervals from network meta-analysis of HAMD score change of individual pharmacotherapy. Drugs are reported in order of efficacy ranking according to SUCRAs. Comparisons should be read from left to right. The efficacy estimate is located at the intersection of the column-defining treatment and the row-defining treatment. For efficacy (mean overall change in symptoms), an MD below 0 favours the column-defining treatment. To obtain MDs for comparisons in the opposing direction, negative values should be converted into positive values and vice versa. Significant results are in bold and underlined. FEWP = Free and Easy Wanderer Plus (a kind of traditional Chinese medicine; its original Chinese name is Jia-Wei-Xiao-Yao-San).

The results of NMA for our primary outcome are presented in Fig. 3A. The ranking of interventions based on surface under the cumulative ranking curve (SUCRA) is presented in Appendix 8. NMA suggested that compared with the control treatment, noradrenaline reuptake inhibitors (NRIs) were associated with a more significant improvement for overall change in the HAMD score [MD 7.90, 95% credible intervals (CrI) 1.91–13.74; SUCRA = 0.85], followed by TCAs (MD 7.64, 95% CrI 3.89–11.07; SUCRA = 0.67), psychotherapy plus antidepressants (P + A) (MD 7.29, 95% CrI 0.02–14.57; SUCRA = 0.62), and SSRIs (MD 6.27, 95% CrI 2.66–9.69; SUCRA = 0.52).

Results for secondary outcomes of patient response rate suggested that rTMS (OR 9.98, 95% CrI 4.06–27.96; SUCRA = 0.86), TCAs (OR 8.01, 95% CrI 4.16–15.42; SUCRA = 0.81), nimodipine plus antidepressants (N + A) (OR 4.80, 95% CrI 1.90–11.74; SUCRA = 0.54), traditional Chinese medicine (TCM) (OR 3.48, 95% CrI 1.62–7.89; SUCRA = 0.50), SSRIs (OR 3.55, 95% CrI 1.98–6.46; SUCRA = 0.40), and P + A (OR 2.35, 95% CrI 1.32–4.45; SUCRA = 0.34) were significantly more effective than the control treatment. rTMS (OR 4.18, 95% CrI 1.38–13.38) and TCAs (OR 3.36, 95% CrI 1.44–8.00) were associated with remarkably higher odds of response rate than P + A. Moreover, TCAs (OR 2.25, 95% CrI 1.20–4.37) were associated with significantly higher odds of response rate than SSRIs (Fig. 3B).

In terms of patient remission rate, rTMS (OR 12.08, 95% CrI 3.03–85.08; SUCRA = 0.84), TCAs (OR 7.55, 95% CrI 2.46–24.94; SUCRA = 0.69), N + A (OR 7.97, 95% CrI 2.12–32.36; SUCRA = 0.67), SSRIs (OR 3.24, 95% CrI 1.30–8.79; SUCRA = 0.38), and P + A (OR 2.25, 95% CrI 1.17–4.54; SUCRA = 0.34) were significantly more effective than the control treatment. In addition, we found that rTMS (OR 5.44, 95% CrI 1.19–41.14) was significantly superior to P + A and TCA (OR 2.31, 95% CrI 1.18–4.83) was significantly superior to SSRIs (Fig. 3B).

### Subgroup and post-hoc sensitivity analyses

With respect to the subgroup of pharmacological interventions, we repeated all the Bayesian NMAs using primary and secondary outcomes as endpoints. The results of the subgroup analysis for the primary outcome are presented in Fig. 3C and Appendix 9. We found that paroxetine (MD 13.40, 95% CrI 3.98–23.34; SUCRA = 0.91), imipramine (MD 11.43, 95% CrI 2.03–21.10; SUCRA = 0.70), reboxetine (MD 9.17, 95% CrI 1.63–16.60; SUCRA = 0.69), nortriptyline (MD 7.85, 95% CrI 3.72–11.88; SUCRA = 0.63), and citalopram (MD 7.74, 95% CrI 9.56–15.18; SUCRA = 0.61) were associated with a significantly better overall change in the HAMD score. In terms of the response rate, nortriptyline was associated with significantly better outcomes than fluoxetine (OR 7.18, 95% CrI 1.14–68.40) and the control (OR 10.02, 95% CrI 1.82–52.56). For the remission rate, citalopram was significantly more effective than paroxetine (OR 3.97, 95% CrI 1.29–12.04). Citalopram (OR 18.50, 95% CrI 2.75–149.70) and paroxetine (OR 4.57, 95% CrI 1.00–28.00) had superior remission rates than the control. The post-hoc sensitivity analysis, including studies using scales such as Montgomery–Asberg Depression Rating Scale (MADRS) and Beck Depression Inventory (BDI), did not change the results appreciably. The ranking was broadly consistent with our previous results. We found that the primary outcome did not change our initial results (NRIs, TCAs, P + A, and SSRIs were proved to be more effective than the control, and the ranking was consistent with our previous results). For the subgroup analysis, fluoxetine became significantly better than the control (sensitivity analysis MD 5.25, 95% CrI 0.40–9.72 vs. main analysis MD −4.56, 95% CrI −0.28 to 9.40). In addition, sertraline ranked worst and remained insignificant.

## Discussion

Our NMA provides a comprehensive synthesis of the available pharmacological and non-pharmacological interventions for PSD. Our results are consistent with those of several reviews^12–14,17^ in demonstrating that SSRIs and TCAs significantly reduce the HAMD score than the control treatment. Interestingly, we conclude that reboxetine can improve the mood status of patients with PSD. Another novel finding is that TCAs appear to be superior to SSRIs and P + A in terms of response and remission rates. Furthermore, we found that rTMS is a beneficial therapeutic approach with regard to response and remission rates compared with the control treatment. In the pharmacological subgroup analysis, paroxetine, citalopram, imipramine, and nortriptyline proved to have a therapeutic benefit with respect to reducing depressive symptoms compared with the control treatment. In addition, we found that nortriptyline is superior to fluoxetine and the control in terms of treatment response.

An unexpected finding was that reboxetine, the first NRI used to treat depression^17^, appears to effectively reduce the HAMD score in PSD patients. Preclinical and clinical observations indicated that reboxetine has high affinity and selectivity for norepinephrine (NE) transporters^17–19^. A review suggested that with its remarkable selectivity to NE over serotonin transporters, reboxetine is a rational alternative for patients who are resistant to conventional antidepressants, such SSRIs and TCAs^17^. Moreover, unlike TCAs, reboxetine shows minimal cardiovascular risk^17^. However, our finding contrasts that of a meta-analysis focused on depression in the general population^20^. The authors argue that reboxetine has little effect in the treatment of major depression^20^. In our NMA, only two trials on reboxetine focused on community settings, which were conducted by the same author^21,22^. This group classified PSD into “retarded” and “anxious” subtypes and suggested that reboxetine is a more effective treatment for “retarded” PSD. However, a few of the classified cases may result in bias. Indeed, we have low confidence with respect to the results of these trials because of the risk of bias and indirect evidence for reboxetine vs. placebo and reboxetine vs. citalopram comparisons. Therefore, these findings do not recommend any treatment, and more RCTs on reboxetine are needed.

A thorough review of related literature revealed that our NMA is the first to show that active rTMS is a beneficial therapeutic approach for managing PSD with respect to response and remission rates compared with P + A or the control treatment. Although the effect of rTMS on the change in the HAMD score is not statistically significant in our NMA, we observed a beneficial trend compared with that observed using the control treatment. This result may indicate that rTMS is favorable for patients with PSD returning to normal mood status. Given that the significance of a simple reduction in mood scores is limited in clinical practice, a good response to treatment or a complete remission of depressive symptoms is arguably the most meaningful outcome for each patient^13^. Moreover, three trials on rTMS recruited patients who were unresponsive to antidepressants given in adequate doses and for at least one course^23–25^, possibly resulting in some heterogeneities. However, these studies clarified the therapeutic effectiveness of rTMS in light of response and remission rates even for patients with drug resistance.

Evidence has shown that P + A is superior to psychotherapy or medication alone, particularly for recurrent depression in older patients^26^. However, no meta-analysis had investigated the effectiveness of this combined therapy particularly for patients with PSD. Hence, our study represents the first comprehensive analysis to demonstrate the superiority of the combined therapy over control therapy in improving mood status of patients with PSD. It should be noticed that the trials concerning “P + A” used different antidepressants, while SSRIs were the most frequently used (details are shown in the appendices). Although some patients with PSD may respond to antidepressants alone, psychotherapy seems to offer additional benefit to treatment success. More research, including cost-effectiveness analyses, is needed to support this hypothesis.

The included trials have inconsistently reported adverse effects. We did not investigate the ranking of the acceptability of outcomes because only a few trials reported these data. However, we noted the different adverse effects of pharmacological and non-pharmacological interventions and summarized them (details are shown in the appendices). The common adverse effects of medications in the treatment group were central nervous system (e.g., headache, sedation, tremor, and fatigue), gastrointestinal, and vascular (e.g., dizziness and palpitation) symptoms. Furthermore, the most common adverse effect of the rTMS therapy was local headache.

Our NMA is based on a small number of studies. Although it indicated that TCAs are superior in terms of efficacy, their anticholinergic effects (e.g., glaucoma, confusion, and urinary retention) and antiadrenergic activity (e.g., hypotension and dizziness) would not make them a first-line treatment^12,27,28^. However, the benefits of nortriptyline on the response rates of patients with PSD should not be ignored. A study has shown that continued use of medication even if the patient does not respond to treatment after 6 weeks has no clear benefit, suggesting that the medication should be changed after this interval^29^. Therefore, to balance the potential benefits and risks, clinicians should consider prescribing TCAs with careful observation when a patient is not responsive to medication after 6 weeks. Besides, although paroxetine is the best treatment in our subgroup analysis, some of its side effects would prevent us to clinically conclude its superiority in patients with stroke. SSRIs have the most anticholinergic effects, which could be expected with their regular use, particularly in the elderly^27,28^. Another particular treatment-emergent symptom of paroxetine is sexual dysfunction (e.g., reduced desire and orgasm dysfunction)^30^. Thus, clinicians should use paroxetine cautiously in patients with stroke, particularly in those with cognitive confusion and compromised sexual function.

Several drawbacks should be noted in the present study. First, risk of bias and methodological deficiencies within individual studies, and the small number of trials in each subgroup of treatments in some nodes may limit our findings for clinical decisions. Hence, although our NMA currently presents the best available evidence, it may not be considered the best possible evidence. Thus, our findings are not decisive and more high-quality RCTs that focus on the duration of PSD are needed. Second, we only retrieved trials that used the HAMD scale to reduce heterogeneity. Although the post-hoc sensitivity analysis justified that excluding studies that used MADRS or BDI did not introduce bias, it still may result in selective bias to some extent. Furthermore, the accuracy of our results may be affected by missing data in non-English trials and negative results of unpublished trials. For example, Ponzio *et al*.’s unpublished trial did not find the superior efficacy of paroxetine^31^, whereas a Hungarian article concluded that paroxetine was well tolerated and effectively improved depressive symptoms^32^. Third, the variable characteristics of patients between trials is a disadvantage. In most studies, participants already on depression treatment were not excluded and only required to stop their antidepressants before participating in the trial. One exception of note is a study by Lipsey *et al*., who only recruited patients not treated with antidepressants^33^. Fourth, several studies excluded patients with communication deficits, cognitive impairment, or previous psychiatric illness, and such criteria probably limit the external validity. Such exclusion criteria may prevent almost half of stroke survivors from partaking in the trial, and the remaining half who were able to participate were probably not representative of patients that require clinical treatment in the “real world”^34,35^. Fifth, given the scarce data, we were unable to treat alternative dosing or duration schemes of the same drug as different nodes in the network, preventing us from investigating potential dose–response and duration–response associations. Sixth, we were unable to quantitatively analyze the safety profile of treatments because of differential reporting of side effects across individual trials and inaccessibility of primary data, which limits clinical application. Seventh, the wide range of years in which the trials were conducted (1984–2016) might introduce heterogeneity. Finally, a few studies only reported per-protocol analyses, which may lead to exaggeration of treatment effects.

To maximize the therapeutic benefits of PSD treatment in pharmacotherapy trials, a key requirement is to choose the appropriate therapeutic dose of the antidepressant for an adequate duration. In addition, future studies should focus on long-term effectiveness and acceptability and perform subgroup analyses based on the length of time between the first appearance of depression and stroke onset. Indeed, depression that occurs in the early stage of stroke seems to be different from that after several months or years of stroke. For psychotherapy trials, evidence has shown that efficacy is associated with adequate exposure to the therapy and the specific therapeutic model. Thus, a standardized framework for therapy, well-trained therapists, and supervision in the pre-specified therapy are needed to achieve desirable results. In each patient with PSD, clinicians should consider the individual clinical profile to balance the potential risks and benefits on a case-by-case basis.

## Methods

This systematic review is reported according to PRISMA statement extension for NMA (Appendix 1)^36^ and conducted according to a priori-established protocol registered with PROSPERO (CRD42016049049)^37^.

### Study selection

#### Criteria for considering studies for this review

Types of studies: We only included RCTs using the HAMD scale for assessing the degree of depression in patients, with data of score change between pre- and post- treatment, or response (defined as at least a 50% reduction in HAMD score) and remission (defined as no longer meeting the baseline criteria for depression) rates to the treatment^13^.

Types of participants: (a) Adults 18 years or older, (b) a clinical diagnosis of ischemic or hemorrhagic stroke, and (c) a clinical diagnosis of PSD based on specific criteria (e.g., DSM- III, DSM-III-R, and DSM-IV) or depression scales (e.g., HAMD scale).

Types of interventions: Interventions comprised pharmaceutical agents (at licensed dose of these medications, alone or in combination with other agents), psychological therapy, ECT, active rTMS, acupuncture therapy, social support, or a combination of these therapies. Specific pharmacological agents include antidepressants [including SSRIs, TCAs, monoamine oxidase inhibitors (MOIs), NRIs, serotonin–noradrenaline reuptake inhibitors (SNRIs)], and TCM. Psychotherapy includes cognitive therapy, behavioral therapy, counseling, problem-solving therapy, and other specific psychosocial programs that help patients improve their emotional status. Control groups include patients with drug placebo, sham or attention control procedures, usual care, and no treatment.

Comparison and outcomes: We analyzed antidepressants according to their substance class (e.g., fluoxetine belongs to SSRIs) and categorized pharmacological interventions into groups: SSRIs, TCAs, NRIs, SNRIs, MOIs, and TCMs. Moreover, we performed analysis on single pharmacological agents (e.g., fluoxetine) as our subgroup outcome. We regarded the mean change in the HAMD score between baseline and endpoint as our primary and subgroup outcomes. For trials that included multiple outcome timepoints, we gave priority to the timepoint of treatment duration used in each trial as endpoint of the study (e.g., treatment duration was 9 weeks, while follow-up lasted 2 years). Secondary outcomes involved response and remission rates.

Furthermore, we performed a post-hoc sensitivity and subgroup analyses for our primary outcome:Then, we conducted an NMA to determine the primary outcome of studies that use MADRS and BDI; these studies had to be excluded from the main analysis because they led to inconsistency. The post-hoc sensitivity analysis aimed to show a more comprehensive evidence of the efficacy of PSD treatments and to justify that exclusion of studies that used MADRS or BDI to obtain the primary outcome did not introduce bias.

Data sources and searches: To compare different PSD treatment strategies, we identified RCTs published in English until November 1, 2016. RCTs were collected from the following databases: PubMed, Embase, and the Cochrane Library Central Register of Controlled Trials. We manually checked relevant meta-analyses in the discipline as well as the reference lists of retrieved publications. A search strategy for each database was adapted (Appendix 2). Two independent investigators (LHD and SQ) initially screened the study titles and abstracts.

Data extraction and quality assessment: Three reviewers (LHD, SQ, and YX) extracted the relevant information from the included trials using a predefined data extraction sheet. An approximation of the mean was used to evaluate the outcomes, where data were merely available in graphic format. The highest standard deviations in the HAMD scores from other trials were retrieved when data were presented without standard deviations^38^.

Data synthesis and statistical analysis: A pairwise meta-analysis applying random-effects model was performed initially^39^. We estimated relative curative effects of the competing interventions using MD for continuous outcomes and OR for dichotomous outcomes, both with 95% CI. The statistical heterogeneity among studies was assessed by Cochran’s Q test and I^2^ statistic. A P value of 0.05 or less for the Q test or an I^2^ greater than 50% indicates substantial study heterogeneity.

For indirect and mixed comparisons, we conducted random-effects Bayesian NMA using Markov chain Monte Carlo methods in WinBUGS version 1.4.3, which use informative prior distributions for all treatment effects as well as the between-study variance parameter^40^. The results of NMA with effect sizes (MD or OR) and CrI were summarized. The pooled estimates were obtained using the Markov chain Monte Carlo method. Three Markov chains were run synchronously with various arbitrarily chosen initial values. We estimated the relative ranking probability of each strategy and obtained the hierarchy of competing interventions using a rankogram, SUCRA^41^.

Furthermore, the loop-specific approach was implemented to check for inconsistency, by assessing the diversity between direct and indirect estimates for a specific comparison in the loop^42^. We employed the node-splitting method, excluding one direct comparison at a time and estimating the indirect treatment effect for the excluded comparison. Then, the design-by-treatment model was used to check for the assumption of consistency^43^. Finally, subgroup analyses were performed to evaluate the robustness of the findings.

Risk of bias and quality of evidence. The validity of the meta-analysis was assessed by qualitative appraisal of study designs and methods. We assessed risk of bias using the Cochrane Collaboration Handbook^38^, focusing on selection, information, and analytical biases. We used the funnel plot to detect publication bias, only when at least 10 studies were available^38^. GRADE was used to evaluate the estimated quality of evidence derived from NMA. In this approach, direct evidence from RCTs starts at a high-quality level, which can be downgraded based on risk of bias, imprecision, indirectness, inconsistency (or heterogeneity), and publication bias to moderate-, low-, and relatively low-quality levels^44^.

## Conclusions

The present study used randomized trial data and a novel evidence synthesis approach, and based on moderate quality evidence, it indicated that SSRIs, TCAs, and NRIs are associated with a significantly reduced HAMD score compared with the control treatment. With regard to response and remission rates, rTMS is a beneficial therapeutic approach for managing PSD and may even be superior in efficacy to SSRIs. In the subgroup analysis, paroxetine, citalopram, nortriptyline, and imipramine proved to be associated with improvement in the HAMD score than the control treatment. However, more high-quality RCTs should be conducted in this field. Future studies should focus on long-term effectiveness and acceptability of treatments and investigate the optimal timing and thresholds of treatments associated with the highest response and remission rates in patients with PSD.

## Electronic supplementary material

Supplementary Information
