# Supplementary material for: Interventions for management of post-stroke depression: A Bayesian network meta-analysis of 23 randomized controlled trials
Source: Sci Rep. 2017 Nov 28;7:16466. doi: 10.1038/s41598-017-16663-0 (PMC5705605; doi:10.1038/s41598-017-16663-0)
Supplement: Supplementary file 1 — Supplementary Information [file 41598_2017_16663_MOESM1_ESM.pdf]

Additional file : Appendices 1-10

Interventions for management of post-stroke depression: A Bayesian network meta-analysis of 23 randomized controlled trials

Linghui Deng[1*], Xuejun Sun[2*], Shi Qiu[3*], Yao Xiong[1], Yuxiao Li[1], Lu Wang[1], Qiang Wei[3], Deren Wang[1#], Ming Liu[1#]

* LHD, XJS and SQ contributed equally as the first authors of this manuscript.

1.Stroke Clinical Research Unit, Department of Neurology, West China Hospital, Sichuan University, Chengdu, Sichuan. China.

2.Second Department of Psychiatry, Kangning Hospital, Anshan, Liaoning, China

3.Department of Urology, Institute of Urology, West China Hospital, Sichuan University, Chengdu, Sichuan, China.

#Correspondence to:

[#]Dr Ming Liu (full professor), Stroke Clinical Research Unit, Department of Neurology, West China Hospital, Sichuan University, Chengdu, Sichuan. China. E-mail: wyplmh@hotmail.com

OR

[#]Dr Deren Wang, Stroke Clinical Research Unit, Department of Neurology, West China Hospital, Sichuan University, Chengdu, Sichuan. China; wdr0128@qq.com

Additional file 1: Appendices 1-10

Abbreviations

Appendix 1: PRISMA network meta-analysis checklist

Appendix 2: Search algorithms

Appendix 3: References for included trials

Appendix 4: Description of included studies, outcomes

Appendix 5: Risk of bias assessments within studies

Appendix 6: Network plot for each outcome

Appendix 7: Assessment of inconsistency

Appendix 8: Treatment ranking and SUCRA plot for each outcome

Appendix 9: Subgroup analysis

Appendix 10: Post hoc analysis

Abbreviations

PSD= post-stroke depression

HAMD= Hamilton Depression Rating Scale(or HRSD)；

DSM= Diagnostic and Statistical Manual of Mental Disorders

RCT= traditional Chinese medicine

SSRI= selective serotonin reuptake inhibitor

TCA= tricyclic antidepressant

SNRI= serotonin–norepinephrine reuptake inhibitors

NRI= norepinephrine reuptake inhibitor

N+A= nimodipine plus antidepressants

TCM= traditional Chinese medicine

rTMS= Repetitive Transcranial Magnetic Stimulation

P+A= psychotherapy plus antidepressants

FEWP=Free and Easy Wanderer Plus (a kind of Chinese medicine; its original Chinese Name is Jia-Wei-Xiao-Yao-San)

MMSE= Mini-mental State examination;

NR=not reported

SD= standard deviation

ITT = intention-to-treat

PP= per protocol

BDI=Beck Depression Inventory

CT=computed tomography

MRI=magnetic resonance imaging

SAH=subarachnoid hemorrhage

TIA=transient ischemic attack

TCD=total cumulative dose

Appendix 2: Search algorithms

Medline	
**#1**	“randomized controlled trial” [Publication Type]
**#2**	“controlled clinical trial” [Publication Type]
**#3**	“randomized” [Title/Abstract]
**#4**	“randomly” [Title/Abstract]
**#5**	“trial” [Title]
**#6**	“Randomized Controlled Trial as Topic” [MeSH]
**#7**	(#1) OR (#2) OR (#3) OR (#4) OR (#5) OR (#6)
**#8**	“PSD” [Title/Abstract]
**#9**	“post-stroke depression” [Title/Abstract]
**#10**	“post-stroke depressive”[Title/Abstract]
**#11**	“depression after stroke”[Title/Abstract]
**#12**	“depression in stroke patients”[Title/Abstract]
**#13**	“depression after cerebral apoplexy”[Title/Abstract]
**#14**	“depression after cerebrovascular accident”[Title/Abstract]
**#15**	“depression after cerebrovascular disease”[Title/Abstract]
**#16**	(#8) OR (#9) OR (#10) OR (#11) OR (#12) OR (#13) OR (#14) OR (#15)
**#17**	“Therapeutics”[Mesh] OR “Antidepressive Agents” [Mesh]
**#18**	“Serotonin Uptake Inhibitors” [Mesh] OR “Fluoxetine” [Mesh] OR “Sertraline” [Mesh] OR “Paroxetine” [Mesh] OR “Citalopram” [Mesh] OR “Fluvoxamine” [Mesh] OR “Escitalopram” [Title/Abstract]
**#19**	“Antidepressive Agents, Tricyclic” [Mesh] OR “Nortriptyline” [Mesh] OR “Imipramine” [Mesh] OR “Clomipramine” [Mesh] OR “Amitriptyline” [Mesh]
**#20**	“serotonin norepinephrine reuptake inhibitor” [Title/Abstract] OR “SNRI” [Title/Abstract] OR “Venlafaxine” [Mesh] OR “Duloxetine” [Mesh] OR “NRI” [Title/Abstract] OR “reboxetine” [Title/Abstract]
**#21**	“Monoamine Oxidase Inhibitors” [Mesh] OR “Methylphenidate” [Mesh] OR “aniracetam” [Title/Abstract] OR “psychostimulant” [Title/Abstract]
**#22**	“Drugs, Chinese Herbal” [Mesh] OR “Acupuncture Therapy” [Mesh]

**#23**	"Psychotherapy" [Mesh] OR "Behavior Therapy" [Mesh]
**#24**	"Transcranial Magnetic Stimulation" [Mesh] OR "Electroconvulsive Therapy" [Mesh]
**#25**	"Mindfulness" [Mesh] OR "Music Therapy" [Mesh] OR "General Surgery" [Mesh] OR "Rehabilitation" [Mesh] OR "Social Support" [Mesh] OR "Education" [Mesh] OR "Family" [Mesh] OR "Nurses" [Mesh]
**#26**	(#17) OR (#18) OR (#19) OR (#20) OR (#21) OR (#22) OR (#23) OR (#24) OR (#25)
**#27**	(#7) AND (#16) AND (#26)
**232**	

**Embase**	
**#1**	'randomized controlled trial'/exp: ti,ab,kw
**#2**	'randomized controlled trial (topic)'/exp: ti,ab,kw
**#3**	random *: ti,ab,kw
**#4**	#1 OR #2 OR #3
**#5**	'PSD'/exp: ti,ab,kw
**#6**	'post-stroke depression'/exp: ti,ab,kw: ti,ab,kw
**#7**	'post-stroke depressive'/exp: ti,ab,kw
**#8**	'depression after cerebral apoplexy'/exp: ti,ab,kw
**#9**	'depression after cerebrovascular accident'/exp: ti,ab,kw
**#10**	'depression after cerebrovascular disease'/exp: ti,ab,kw
**#11**	'depression in stroke patients'/exp: ti,ab,kw
**#12**	'depression after stroke' /exp: ti,ab,kw
**#13**	#5 OR #6 OR #7 OR #8 OR #9 OR #10 OR #11 OR #12 OR
**#14**	'therapeutics' /exp: ti,ab,kw OR 'antidepressive Agents' /exp: ti,ab,kw
**#15**	'tricyclic' /exp: ti,ab,kw
**#16**	'serotonin uptake inhibitors' /exp: ti,ab,kw
**#17**	'serotonin norepinephrine reuptake inhibitor' /exp: ti,ab,kw
**#18**	'monoamine oxidase inhibitors' /exp: ti,ab,kw
**#19**	'fluoxetine' /exp: ti,ab,kw OR 'sertraline' /exp: ti,ab,kw OR 'paroxetine' /exp: ti,ab,kw OR 'citalopram' /exp: ti,ab,kw
**#20**	'Chinese Herbal' /exp: ti,ab,kw OR 'acupuncture' /exp: ti,ab,kw

**#21**	‘psychotherapy’ /exp: ti,ab,kw OR ‘behavior therapy’ /exp:ti,ab,kw OR ‘transcranial magnetic stimulation’ /exp: ti,ab,kw OR ‘electroconvulsive therapy’ /exp: ti,ab,kw
**#22**	‘mindfulness’ /exp: ti,ab,kw OR ‘music therapy’ /exp: ti,ab,kw OR ‘surgery’ /exp: ti,ab,kw OR ‘rehabilitation’ /exp: ti,ab,kw OR ‘education’ /exp: ti,ab,kw
**#23**	(#14) OR (#15) OR (#16) OR (#17) OR (#18) OR (#19) OR (#20) OR (#21) OR (#22)
**#24**	(#4) AND (#13) AND (#23)
**318**	

**Cochrane Library Central**	
**#1**	PSD: ti, ab, kw OR post- stroke depression: ti, ab, kw in Trials (Word variations have been searched)
**#2**	depression after cerebrovascular disease: ti, ab, kw OR depression after cerebral apoplexy: ti, ab, kw in Trials (Word variations have been searched)
**#3**	depression after stroke: ti, ab, kw in Trials (Word variations have been searched)
**#4**	depression in stroke patients : ti, ab, kw in Trials (Word variations have been searched)
**#5**	(#1) OR (#2) OR (#3) OR (#4)
**#6**	therapeutics: ti, ab, kw OR antidepressive: ti, ab, kw OR serotonin reuptake inhibitor: ti, ab, kw OR tricyclic: ti, ab, kw OR monoamine oxidase inhibitor: ti, ab, kw in Trials (Word variations have been searched)
**#7**	fluoxetine: ti, ab, kw OR sertraline: ti, ab, kw OR paroxetine: ti, ab, kw OR citalopram: ti, ab, kw OR reboxetine: ti, ab, kw in Trials (Word variations have been searched)
**#8**	trazodone: ti, ab, kw OR nortriptyline: ti, ab, kw OR escitalopram: ti, ab, kw OR psychostimulant: ti, ab, kw in Trials (Word variations have been searched)
**#9**	Chinese herbal medicine: ti, ab, kw OR acupuncture: ti, ab, kw OR behavior therapy: ti, ab, kw OR psychotherapy: ti, ab, kw in Trials (Word variations have been searched)
**#10**	transcranial magnetic stimulation: ti, ab, kw OR surgery: ti, ab, kw OR electroconvulsive therapy: ti, ab, kw OR mindfulness: ti, ab, kw OR music therapy: ti, ab, kw in Trials (Word variations have been searched)
**#11**	nurse: ti, ab, kw OR care: ti, ab, kw OR support: ti, ab, kw OR family: ti, ab, kw OR education: ti, ab, kw in Trials (Word variations have been searched)
**#12**	(#6) OR (#7) OR (#8) OR (#9) OR (#10) OR (#11)
**#13**	(#5) AND (#12)
**602**	

Appendix 3: References for included trials

1. Lipsey JR, Robinson RG, Pearlson GD, Rao K, Price TR. Nortriptyline treatment of post-stroke depression: a double-blind study. Lancet. **l**, 297-300 (1984).
2. Andersen G, Vestergaard K, Lauritzen L, General F. Original contributions effective treatment of post-stroke depression with the selective serotonin reuptake inhibitor citalopram. Stroke. 1994;25(6):1099-1104.
3. Gonzalez-torrecillas JL, Mendlewicz J, Lob A. Effects of early treatment of post-stroke depression on neuropsychological rehabilitation. Int Psychogeriatr. **7**, 547-560 (1995)
4. Kimura M, Robinson RG, Kosier JT. Treatment of cognitive impairment after post-stroke depression: A double-blind treatment trial. Stroke. **31**, 1482–1486 (2000).
5. Robinson RG, Schultz SK, Castillo C, Kopel T, Kosier JT, Newman RM, et al. Nortriptyline versus fluoxetine in the treatment of depression and in short-term recovery after stroke. Am J Psychiatry. **157**, 351–359 (2000).
6. Taragano FE, Allegri R, Vicario A, Bagnatti P, Lyketsos CG. A double blind, randomized clinical trial assessing the efficacy and safety of augmenting standard antidepressant therapy with nimodipine in the treatment of 'vascular depression'. Int J Geriatr Psychiatry. **16**, 254–260 (2001).
7. Fruehwald S, Gatterbauer E, Rehak P, Baumhackl U. Early fluoxetine treatment of post-stroke depression: A three-month double-blind placebo-controlled study with an open-label long-term follow up. J Neuro. **250**, 347–351 (2003).
8. Kimura M, Tateno A, Robert G, Robinson M. Treatment of post-stroke generalized anxiety disorder comorbid with post-stroke depression merged analysis of nortriptyline trials. Am J Geriatr Psychiatry. **11**, 320–327 (2003)
9. Rampello L, Chiechio S, Nicoletti G, Alvano A, Vecchio I, Raffaele R. Prediction of the response to citalopram and reboxetine in post-stroke depressed patients. Psychopharmacology. **173**, 73–78 (2004).
10. Rampello L, Alvano A, Chiechio S. An evaluation of efficacy and safety of reboxetine in elderly patients affected by "retarded" post-stroke depression: A random, placebo-controlled study. Arch Gerontol Geriatr. **40**, 275–285 (2004).
11. Huang L. Comparison of therapeutic effects and side effects between fluoxetine hydrochloride and clomipramine in patients with vascular depression. Chin J Clin Rehabil. **9**, 226–228 (2005).
12. Ye LX, Wang YD, Wang H, Liang DS. Effect of Paxil and berhomine on post-stroke anxiety-depression and neurological recovery. Chin J Clin Rehabil. **10**, 153-155 (2006).
13. Li LT, Wang SH, Ge HY, Chen J, Yue SW, Yu M. The beneficial effects of the herbal medicine Free and Easy Wanderer Plus (FEWP) and fluoxetine on post-stroke depression. J Altern Complement Med. **14**, 841–846 (2008).
14. Cravello L, Caltagirone C, Spalletta G. The SNRI venlafaxine improves emotional unawareness in patients with post-stroke depression. Hum. Psychopharmacol Clin Exp. **24**, 331–336 (2009).
15. Karaiskos D, Tzavellas E, Spengos K, Vassilopoulou S, Paparrigopoulos T. Duloxetine versus citalopram and sertraline in the treatment of post-stroke depression, anxiety, and fatigue. J Neuropsychiatry Clin Neurosci. **24**,349–353 (2012).
16. Jorge RE, Robinson RG, Tateno A, Narushima K, Acion L, Moser D, et al. Repetitive transcranial magnetic stimulation as treatment of post-stroke depression: A preliminary study. **55**, 398–405 (2004).
17. Jorge RE. Moser DJ, Acion L, Robinson RG. Treatment of vascular depression using repetitive transcranial magnetic stimulation. Arch Gen Psychiatry. **65**, 268-276 (2008).
18. Tenev V, Robinson RG, Jorge RE. Citalopram for continuation therapy after repetitive transcranial magnetic stimulation in vascular depression. Am J Geriatr Psychiatry.**17**, 682–687 (2010).
19. Narushima K, McCormick L, Yamada T, Thatcher R, Robinson RG. Subgenual cingulate theta activity predicts treatment response of repetitive transcranial magnetic stimulation in participants with vascular depression. J Neuropsychiatry Clin Nerosci. **22**, 75-84 (2010).
20. Seo YG, Chang MC. The Effects of 10-Hz Repetitive Transcranial Magnetic Stimulation on Depression in Chronic Stroke Patients. [published online ahead of print October 18, 2016]. Brain Stimul. http://www.brainstimjrnl.com/article/S1935-861X(16)30270-4/abstract. Accessed September 9, 2016.
21. Feng SZ, Zhang MY, Dai AH. Impacts of rehabilitative therapy on post-stroke depression and the ability of daily life. Chin J Clin Rehabil. **9**, 154-155 (2005).
22. Williams LS, Kroenke K, Bakas T, Plue LD, Brizendine E, Hendrie H. Care Management of Poststroke Depression: A Randomized, Controlled Trial. Stroke. **38**, 998–1003 (2007).

23. Mitchell PH. Brief psychosocial-behavioral intervention with antidepressant reduces poststroke depression significantly more than usual care with antidepressant: living well with stroke: randomized, controlled trial. Stroke. **40**, 3073–3078 (2009).

24. Wiart L, Petit H, Joseph PA, Mazaux JM, Barat M. Fluoxetine in early post-stroke depression: a double-blind placebo-controlled study. Stroke. **31**, 1829–32 (2000).

25. Murray V, Von ArbinM, Bartfai A, Berggren A, Landtblom A, Lundmark J, Martensson B. Double-blind comparison of sertraline and placebo in stroke patients with minor depression and less severe major depression. Journal of Clinical Psychiatry. **66**, 708-716 (2005)

Appendix 4: Description of included studies, outcomes

Table1. Study characteristic

study	Inclusion criteria	Exclusion criteria				profile of prior antidepressant therapy
		diseases limiting verbal comprehension[a]	psychiatric illness or substance abuse	CNS diseases	others	
Lipsey 1984	thromboembolic stroke or intracerebral hemorrhage; moderate or severe depression; with informed consent	severe comprehension deficit	-	-	medical contraindication to nortriptyline	only included patients never being treated with antidepressants
Andersen 1994	acute stroke with depression	decreased consciousness; aphasia; dementia	history of psychiatric illness (except depression more than 1 year earlier)	SAH; Binswanger's disease; previous degenerative or expansive neurological diseases[b]	-	exclude current antidepressant treatment
Gonzalez 1995	unilateral lesion documented by CT scan and capable of compliance were included. with informed consent.	aphasia >2b/3 according to the Goodglass criteria	alcoholism, drug abuse, any pathological condition capable of resembling a depressive condition	-	-	exclude antidepressant treatment in the 6 months before stroke

Robinson 2000	acute stroke within 6 months of the onset of the study and age 18–85	severe comprehension deficit	-	head injury, prior or other brain disease except prior stroke	significant medical illness	patients who taking antidepressants were required to stop the therapy before the study (N=3).
Kimura 2000	acute thromboembolic or intracerebral hemorrhagic infarction who were identified as depressed	decreased consciousness; aphasia; dementia	-	-	-	-
Taragano 2001	With vascular depression , and met DSM-IV criteria for major depressive episode	MMSE<24	-	-	too ill medically	-
Fruehwald 2003	thromboembolic stroke or intracerebral hemorrhage were verified by CT. moderate or severe depression, as measured by a HAMD>15	more than mild communication deficit; MMSE<20	-	previous degenerative or expansive neurological diseases	-	-
Kimura 2003	acute thromboembolic or intracerebral hemorrhagic infarction, and identified as depressed	decreased consciousness; aphasia; dementia	-	-	-	-
Rampello 2003	presence of a recent (<12 months) single ischemic or hemorrhagic stroke, which was documented by CT or MRI; presence of major or minor depression, according to DSM IV criteria, with HDRS>20, BDI> 15	decreased consciousness; severe aphasia, severe cognitive deficit(MMSE <22)	history of psychiatric illness (except depression for more than 1 year); chronic alcoholism,	previous degenerative or expansive neurological diseases, SAH, Binswanger's disease	respiratory complications; serious heart diseases; under anticoagulant treatment	lack of antidepressant treatment within 30 days prior to this study

Rampello 2004	presence of a recent (<12 months) single ischemic or hemorrhagic stroke, (documented by CT or MRI); presence of major or minor depression, according to DSM IV criteria, with HDRS>20 and BDI>15; with informed consent.	severe aphasia, severe cognitive deficit	history of psychiatric illness (other than depression); chronic alcoholism.	previous degenerative or expansive neurologic disease, SAH, Binswanger's disease	-	lack of antidepressant treatment within 2 weeks prior to this study
Huang 2005	with diagnosis of vascular depression; less than 70 years old	decreased consciousness; dementia; severe mental disorders	history of depression	trauma, tumor, inflammation or demyelination in brain	severe impairment in cardiac function, hepatic function or renal function; history of drug allergy	-
Ye 2006	post-stroke anxiety and depression, with HAMD-24>21 and HAMA-14>14	decreased consciousness; understanding problems;	-	-	without stable life signs	-
Li 2008	presence of a recent (<6 weeks) single ischemic or hemorrhagic stroke, documented by CT or MRI; presence of major or minor depression, with a HAMD>20	severe aphasia; MMSE<23	a history of psychiatric illness other than depression; chronic alcoholism	epilepsy	abnormal thyroid function	lack of before the enrolment within 2 weeks prior to this study
Cravello 2009	first-ever stroke diagnosis within the last 12 months and diagnosis of post-stroke major depressive-like episode.	severe cognitive impairment （MMSE<12）	history of psychiatric disorders, within 5 years before the stroke	stroke history; degenerative or expansive neurological diseases	atherosclerotic disease, major medical illnesses	-
Dimitrios 2012	diagnosis of the first-ever stroke within the last 12 months, based on clinical history, physical examination, and findings of brain MRI; Diagnosis of PSD, according to DSM-IV.	dementia; Severe cognitive impairment(MMSE<24)	history of a major psychiatric disorder within 5 years before the stroke.	stroke history; degenerative or expansive neurological diseases	atherosclerotic disease or a history of angioplasty or bypass surgery; major medical illness	-

Jorge 2004	cortical or subcortical ischemic lesions of the right hemisphere and subcortical lesions or posterior cortical lesions of the left hemisphere.	aphasia or with language comprehension deficits; MMSE<23	actively suicidal; active psychosis; bipolar course; alcohol or drug abuse during the past 12 months	degenerative neurological diseases ; major head trauma, epilepsy	severe systemic disease; ongoing neoplasia; contraindications for rTMS[c]	patients were unresponsive to antidepressants
Jorge 2008	major depressive disorder (as diagnosed by DSM-IV criteria) at age 50 years or older, a history of subcortical stroke, and/or at least 3 of the following cardiovascular risk factors: arterial hypertension, diabetes mellitus, obesity, hyperlipidemia, and smoking	dementia	suicidal; active psychosis; comorbid alcohol or other drug abuse within 2 years before the study	degenerative neurological diseases; head trauma, epilepsy	severe heart or respiratory failure; renal or hepatic failure; ongoing neoplasia; contraindications for rTMS	Patients were unresponsive to antidepressants; patients who taking antidepressants were required to stop the therapy before initiation of rTMS
Narushima 2010	age between 50 and 90 years old; major depressive disorder (as diagnosed by the DSM-IV-TR) with HAMD>14; a history of stroke or at least three of the following cardiovascular risk factors: arterial hypertension, diabetes mellitus, obesity, hyperlipidemia, and smoking	severe aphasia; dementia	suicidal thought, plan, or delusion; substance abuse within the prior 2 years;	degenerative neurological diseases; prior seizure or traumatic brain injury	life-threatening physical illness; contraindications for rTMS	patients were unresponsive to antidepressants; patients who taking antidepressants were required to stop the therapy before initiation of rTMS
Tenev 2010	with onset of major depression at age 50 or older, with a history of subcortical stroke or at least 3 of the following cardiovascular risk factors: arterial hypertension, diabetes mellitus, obesity, hyperlipidemia, and smoking	-	psychotic depression	-	severe coexistent medical illness	patients who taking antidepressants were required to stop the therapy before initiation of rTMS
Seo 2016	history of stroke ≥ 6 months; aged between 21 and 80 years; the presence of depression (BDI>12 and HAMD-17>6)	aphasia; severe cognitive dysfunction	depression before stroke onset;	-	serious medical complication; contraindications for rTMS	patients with medication history of antidepressants before stroke onset were excluded
Feng 2004	stroke patients with depression	decreased consciousness; aphasia; cognitive disorder; mental disorder	-	previous stroke history	serious condition	-

Williams 2007	could speak and understand English, had a telephone, and who had a life expectancy of at least 6 months	aphasia; dementia; MMSE<23	active psychosis; suicidality; substance abuse	hemorrhagic stroke	women pregnant at the time of stroke	patients were not excluded for prior antidepressant treatment either before or at the time of the stroke
Mitchell 2009	within 4 months of an ischemic stroke, verified by CT or MRI, who screened positive for depressive symptoms, and whose diagnosis of clinical depression was verified by DSM IV criteria	-	active psychosis; drug abuse	-	-	patients were not excluded for prior or current antidepressant treatment

[a] Diseases limiting verbal comprehension including decreased consciousness, aphasia, dementia, and cognitive disorder.

[b] Degenerative neurological diseases including Parkinson disease and Alzheimer disease; expansive neurological diseases including multiple sclerosis, tumor, hydrocephalus, and amyotrophic lateral sclerosis.

[c] Contraindications for rTMS including epileptic seizure, the presence of metal in the skull, pacemaker placement, frontal cortex lesion or hemorrhagic stroke (to avoid rTMS induced seizure).

HAMD= Hamilton Depression Rating Scale(or HRSD)；

DSM= Diagnostic and Statistical Manual of Mental Disorders

CT=computed tomography

MRI=magnetic resonance imaging

SAH=subarachnoid hemorrhage

TIA=transient ischemic attack

TCD=total cumulative dose

MMSE= Mini-mental State examination;

BDI=Beck Depression Inventory

Table 2. Treatment characteristic

study	duration	intervention/control group (N and maximum daily dose)		
Lipsey 1984	6 weeks	Nortriptyline (N=14;100mg/d)	Placebo (N=20)	-
Andersen 1994	6 weeks	Citalopram (N=33;20mg/d)	Placebo (N=33)	-
Gonzalez 1995	6 weeks	Fluoxetine (N=26;20mg/d)	Nortriptyline (N=11;75mg/d)	Placebo (N=11;100mg/d)
Robinson 2000	12 weeks	Fluoxetine (N=23;40mg/d)	Nortriptyline (N=16,100mg/d	Placebo (N=17)
Kimura 2000	6 or 12 weeks	Nortrityline (N=21;100mg/d)	Placebo (N=26)	-
Taragano 2001	60 days	Nimodipine + antidepressants (N=40,90mg/d)	SSRI (N=44)	-
Fruehwald 2003	12 weeks	Fluoxetine (N=28; 20mg/d);	Placebo (N=26)	-
Kimura 2003	6 or 12 weeks	Nortrityline (N=13;100mg/d)	Placebo (N=14)	-

Rampello 2003	16 weeks	Citalopram<br>(N=37;20mg/d)	Reboxetine<br>(N=37;4mg/d)	-
Rampello 2004	16 weeks	Reboxetine<br>(N=16;4mg/d);	Placebo<br>(N=15)	-
Huang 2005	12 weeks	Fluoxetine<br>(N=30;20mg/d)	Clomipramine<br>(N=30;750mg/d)	-
Ye 2006	12 weeks	Paroxetine<br>(N=29;20mg/d)	Imipramine<br>(N=27;150mg/d)	Control<br>(n=27)
Li 2008	8 weeks	FEWP<br>(N=60;36g/d)	Fluoxetine<br>(N=60;40mg/d)	Placebo<br>(N=30;36g/d)
Cravello 2009	8 weeks	Fluoxetine<br>(N=25;40mg/d)	Venlafaxine (N=25;150mg/d)	-
Dimitrios 2012	3 months	Duloxetine<br>(N=20;120mg/d)	Citalopram<br>(N=20;40mg/d)	Sertraline<br>(N=20;200mg/d)
Jorge 2004	2 weeks	Active rTMS<br>(N=10;TCD=12K[a])	Sham rTMS<br>(N=10)	-
Jorge 2008[c]	3 weeks	Active rTMS<br>(N=15;TCD=12K)<br>(N=33;TCD=18K[b])	Sham rTMS<br>(N=15)<br>(N=29)	-
Narushima 2010	2 weeks	Active rTMS<br>(N=43;TCD=12k/18k)	Sham rTMS<br>(N=22)	-
Tenev 2010	2 weeks	Active rTMS<br>(N=33;TCD=18k)	Sham rTMS<br>(N=29)	-
Seo 2016	2 weeks	Active rTMS<br>(N=12;TCD=12K)	Sham rTMS<br>(N=12)	-

Feng 2004	6 months	Psychotherapy<br>(N=30)	Control<br>(N=30)	-
Williams 2007	12 weeks	P+A[d]<br>(N=89)	Control<br>(N=93)	-
Mitchell 2009	8 weeks	P+A[e]<br>(N=48)	SSRI<br>(N=53)	-

[a]12K=12 000 magnetic pulses.

[b]18K=18 000 magnetic pulses.

[c]The patients were randomized divided into two intervention group: 12K-group and 18K-group

[d]Most patients used SSRI, while some patients used an alternate antidepressant, based on his or her own assessment

[e]Patients used paroxetine, or sertraline (for subjects who had a history of side effects or lack of response to paroxetine), or changed to venlafaxine (for subjects with <50% drop in PHQ-9 score at 6 weeks).

P+A= Psychotherapy plus antidepressants therapy

TCM= traditional Chinese medicine

SSRI=selective serotonin reuptake inhibitor

rTMS= Repetitive Transcranial Magnetic Stimulation

Table 3. Results of individual studies

3-1 reduction of HAMD score between pre- and post- treatment of all treatments

	**SSRI**			**TCA**			**SNRI**			**NRI**			**N+A**			**TCM**			**CONTROL**			rTMS			**P+A**			**Psychotherapy**		
	n	mean	sd	n	mean	sd	n	mean	sd	n	mean	sd	n	mean	sd	n	mean	sd	n	mean	sd	n	mean	sd	n	mean	sd	n	mean	sd
Lipsey 1984				11	11.14	0.69													15	6.39	1.03									
Andersen 1994	33	8	6																33	4.8	4.6									

Gonzalez 1995	25	16.37	7.9	10	14.68	0.35													10	2.11	0.36									
Robinson 2000	14	1.9	6.64	13	13.5	7.47													13	5.3	5.6									
Kimura 2000				18	12.05	5.53													26	6.84	6.76									
Taragano 2001	44	13.08	4.21										40	15.6	5.67															
Fruehwald 2002	26	23.3	12																24	19.1	15.1									
Kimura 2003				13	10.2	8.2													14	2.8	7.4									
Rampello 2003-(retarded)[a]	14	2.58	3.89							15	13.58	2.49																		
Rampello 2003-(anxious)[a]	20	13.95	2.02							19	1.89	3.62																		
Rampello 2004										16	14.8	1.91							15	1.27	2.08									
Huang 2005	30	13.5	3.8	30	12.19	3.64																								
Ye 2006	29	21.16	6.095	27	19.18	7.97													27	7.8	4.591									
Li 2008	58?	10.8	2.81													60	10.6	3.36	28	5.6	3.5									
Cravello 2009	25	9.07	4.17				25	7.33	4.23																					
Dimitrios 2012[b]	40	20.1	6.24				20	21.1	6.87																					
Jorge 2004																			10	2.7	1.51	10	7.3	1.51						

Jorge 2008-(12K)[c]																			15	2.71	1.03	15	6.45	1.03						
Jorge 2008-(18K)[c]																			29	3.08	6.09	33	7.8	4.7						
Narushima 2010																			11	3.8	1.77	32	5.27	2.59						
Seo 2016																			12	-0.3[d]	0.79	12	3.2	1.13						
Feng 2004																			30	-1.2[d]	7.02							30	3.4	4.5
Williams 2006																			93	5.4	8				89	7.4	7.2			
Mitchell 2009	53	3.6	5.6																						45	9.8	4.9			

[a]The author divided PSD patients into “retarded” and “anxious” groups, and in each group the patients were randomized into citalopram and reboxetine subgroups.

[b]Data of SSRI group were pooled from two group: citalopram group (n=20; mean HAMD=21; sd=5.96) sertraline group (n=20; mean HAMD=19.6; sd=6.6)

[c]The anthor divided PSD patients into 2 group according to the total cumulative dose(TCD) the active groups accepted.

[d]Minus represents increase of HAMD score which means the HAMD scores were increased after treatnents.

SSRI= selective serotonin reuptake inhibitor

TCA= tricyclic antidepressant

SNRI= serotonin–norepinephrine reuptake inhibitors

NRI= norepinephrine reuptake inhibitor

N+A= nimodipine plus antidepressants

TCM= traditional Chinese medicine

rTMS= Repetitive Transcranial Magnetic Stimulation

P+A= psychotherapy plus antidepressants

3-2 response of all treatments

	SSRI		TCA		N+A		TCM		CONTROL		rTMS		P+A	
	Response	n	Response	n	Response	n	response	n	Response	n	response	n	response	n
Robinson 2000	2	23	10	16					4	17				
Kimura 2000			16	21					8	26				
Taragano 2001	25	44			27	40								
Fruehwald 2002	18	26							18	24				
Kimura 2003			9	13					3	14				
Huang 2005	25	30	25	30										
Ye 2006	20	29	18	27					7	27				
Li 2008	38	58					36	60	6	28				
Jorge 2004									0	10	3	20		
Jorge 2008-(12K)[a]									1	15	5	15		
Jorge 2008-(18K)[a]									2	29	13	33		
Narushima 2010									1	11	14	32		
Tenev 2010									2	29	13	33		
Williams 2006									28	93			45	89

[a]The anthor divided PSD patients into 2 group according to the total cumulative dose(TCD) the active groups accepted.

SSRI= selective serotonin reuptake inhibitor

TCA= tricyclic antidepressant

N+A= nimodipine plus antidepressants

TCM= traditional Chinese medicine

rTMS= Repetitive Transcranial Magnetic Stimulation

P+A= psychotherapy plus antidepressants

3-3 remission of all treatments

	SSRI		TCA		N+A		CONTROL		rTMS		P+A	
	remission	n	remission	n	remission	n	remission	n	remission	n	remisison	n
Andersen 1994	16	27					9	32				
Taragano 2001	11	44			18	40						
Ye 2006	7	29	6	27			2	27				
Jorge 2004							0	10	1	10		
Jorge 2008-(12K)[a]							1	15	2	15		
Jorge 2008-(18K)[a]							1	29	9	33		
Narushima 2010							0	11	11	32		
Williams 2006							21	93			35	89
Mitchell 2009	10	53									21	45

[a]The anthor divided PSD patients into 2 group according to the total cumulative dose(TCD) the active groups accepted.

SSRI= selective serotonin reuptake inhibitor

TCA= tricyclic antidepressant

N+A= nimodipine plus antidepressants

rTMS= Repetitive Transcranial Magnetic Stimulation

P+A= psychotherapy plus antidepressants

3-4 reduction of HAMD score between pre- and post- treatment of antidepressants

	**SSRI**												**SNRI**						**TCA**									**NRI**			**TCM**			**CONTROL**		
	sertraline			sertraline			fluoxetine			paroxetine			duloxetine			venlafaxine			imipramine			clomipramine			nortriptyline			reboxetione			Free and Easy Wanderer Plus			control		
	n	mean	sd	n	mean	sd	n	mean	sd	n	mean	sd	n	mean	sd	n	mean	sd	n	mean	sd	n	mean	sd	n	mean	sd	n	mean	sd	n	mean	sd	n	mean	sd
Lipsey 1984																									11	11.14	0.69							15	6.39	1.03
Andersen 1994	33	8	6																															33	4.8	4.6
Gonzalez 1995							25	16.37	7.9																10	14.68	0.35							10	2.11	0.36
Robinson 2000							14	1.9	6.64																13	13.5	7.47							13	5.3	5.6
Kimura 2000																									18	12.05	5.53							26	6.84	6.76

Fruehwald 2002							26	23.3	12																									24	19.1	15.1
Kimura 2003																									13	10.2	8.2							14	2.8	7.4
Rampello 2003-(anxious)[a]	20	13.95	2.02																									19	1.89	3.62						
Rampello 2003-(retarded)[a]	14	2.58	3.89																									15	13.58	2.49						
Rampello 2004																												16	14.8	1.91				15	1.27	2.08
Huang 2005							30	13.5	3.8													30	12.19	3.64												
Ye 2006										29	21.16	6.095							27	19.18	7.97													27	7.8	4.591
Li 2008							58	10.8	2.81																						60	10.6	3.36	28	5.6	3.5

Cravello 2009							25	9.07	4.17							25	7.33	4.232																		
Dimitrios 2012	20	20.6	5.96	20	19.6	6.62							20	21.1	6.87																					

[a]The author divided PSD patients into “retarded” and “anxious” groups, and in each group the patients were randomized into citalopram and reboxetine subgroups.

SSRI= selective serotonin reuptake inhibitor

TCA= tricyclic antidepressant

SNRI= serotonin–norepinephrine reuptake inhibitors

NRI= norepinephrine reuptake inhibitor

TCM= traditional Chinese medicine

3-5 response of all antidepressants

	SSRI				TCA						TCM		CONTROL	
	fluoxetine		paroxetine		imipramine		clomipramine		nortriptyline		Free and Easy Wanderer Plus		control	
	Response	n	Response	n	Response	n	response	n	response	n	response	n	Response	n
Robinson 2000	2	23							10	16			4	17
Kimura 2000									16	21			8	26
Fruehwald 2002	18	26											18	24
Kimura 2003									9	13			3	14
Huang 2005	25	30					25	30						
Ye 2006			20	29	18	27							7	27

Li 2008	38	58									36	60	6	28

SSRI= selective serotonin reuptake inhibitor

TCA= tricyclic antidepressant

TCM= traditional Chinese medicine

3-6 remission of antidepressants

	SSRI				TCA		CONTROL	
	citalopram		paroxetine		imipramine		control	
	remission	n	remission	n	remission	n	remission	n
Andersen 1994	16	27					9	32
Ye 2006			7	29	6	27	2	27

SSRI= selective serotonin reuptake inhibitor

TCA= tricyclic antidepressant

Table 4. Overview of treatments in the included trials and in the network meta-analysis

intervention groups	Trial group/arms
TCA, tricyclic antidepressants	7 arms in total<br>5 nortriptyline<br>1 imipramine<br>1 clomipramine
SSRI, selective serotonin reuptake inhibitors	14 separate groups in trials, after pooling of 2 arms in a study with 2 SSRI, 13 arms in analyses<br>4 citalopram (1 pooled with sertraline, 2 from 1 trial[a])<br>1 sertraline (pooled with citalopram)<br>6 fluoxetine

	1 paroxetine<br>2 mixed use of SSRIs
SNRI, serotonin-noradrenaline reuptake inhibitor	2 arms in total<br>1 duloxetine<br>1 venlafaxine
NRI, noradrenaline reuptake inhibitor	3 arms in total<br>3 reboxetine (2 from 1 trial[a])
TCM, Chinese traditional medicine	1 arms in total<br>1 Free and Easy Wanderer Plus
rTMS therapy	6 arms in total (2 from 1 trial[b])<br>3 TCD=12k<br>2 TCD=18k<br>1 TCD=12k or 18k
Psychothetapy	1 arm in total
Nimodipine plus antidepressants	1 arms in total<br>1 nimodipine plus antidepressants
Psychotherapy plus antidepressants	2 arms in total<br>2 psychotherapy plus antidepressants

[a]In trial of "Rampello 2003", the author divided PSD patients into "retarded" and "anxious" groups, and in each group the patients were randomized into citalopram and reboxetine subgroups.

[b]In trial of "Jorge 2008", the anthor divided PSD patients into 2 group according to the total cumulative dose(TCD) the active groups accepted.

Table 5. Adverse events of individual studies

study	Adverse events<br>(Showed as events n(%))		
	Group 1	Group 2	Group 3

Lipsey 1984	nortriptyline: dizziness 1; delirious 3; sedated 1; syncopal 1	placebo: mania 1; refused interview or unfollowed 3; death 2(heart-failure 1; ICH 1); dizziness 1	
Andersen 1994	citalopram: new stroke 3(thromboembolic 1; ICH 1; TIA 1); epilepsy 2; rash 1; death 2(both not because of heart-failure)	placebo: death 2(heart-failure) ; heart-failure 1; acute myocardial infarction 1; new stroke 2(thromboembolic 1;TIA 1); rash 1	
Gonzalez 1995	fluoxetine:1 drop out because of side effects;	nortriptyline:1 drop out because of side effects	control: unfollowed
Robinson 2000	fluoxetine: gastrointestinal symptoms 3; refused treatment 6;	nortriptyline: medical deterioration 2; refused treatment 1	placebo: death 1(pulmonary embolus 1); medical deterioration 1; refused treatment 2
Kimura 2000	NR		
Taragano 2001	nimodipine: hypotention 7; nausea 2; headache 2; dizziness 4;retarded ejaculation 1; epigastric pains 3; bradycardia 1; vertigo 1; insomnia 1; diarrhea 1; any one 21	control: hypotention 1; nausea 5; headache 4; dizziness 1; dry mouth 3; retarded ejaculation 2; bradycardia 1; anorexia 2; phobia 1; bronchitis 1; any one 18	
Fruehwald 2003	fluoxetine: death 1; pulmonary artery embolism 1	placebo: medical deterioration 1(suicidal 1); dermatological disease 1	
Kimura 2003	NR		
Rampello 2003	citalopram: nausea (3%), vomiting (2%), asthenia and fatigability (4%), opening insomnia (18%), weight increase (12%), and reduction of sexual activity (7%).	reboxetine: dry mouth (19%), constipation (16%), hyperperspiration (16%), drowsiness (5%), urinary wavering or urinary retention (4%), hypotension (7%), and sinusal tachycardia (6%)	

Rampello 2004	reboxetine: dry mouth (22%), constipation (18%), hyperperspiration (16%), insomnia (4%),drowsiness (3%), urinary wavering or urinary retention (4%), hypotension (8%), and sinusal tachycardia (7%).	placebo: dry mouth (19%), constipation (15%), hyperperspiration (12%), insomnia (5%),drowsiness (5%), hypotension (2%), and sinusal tachycardia (1%).	
Huang 2005	fluoxetine:8 patients(27%) had adverse events, and 2 of them might be linked to the medication (Nausea, thirsty)	clomipramine:13 patients (43%) had adverse events, 10 of them might be linked to the medication (thirsty, constipation, voiding dysfunction, dizziness and excitation)	
Ye 2006	paroxetine: unfollowed 1; Nausea 2;	imipramine: refused treatment 3 (because of xerostomia, constipation, blurred vison, cardiovascular side effects)	control: death 1(ICH);unfollowed 2
Li 2008	TCM:2 Nausea;	fluoxetine: Nausea 6;insomnia 4; second stroke 2	placebo:3 Nausea; 2 insomnia; 2 aggravated symptoms of depression
Cravello 2009	fluoxetine: insomnia; Nausea, fatigability, cephalalgia, and dizziness(all events were mild)	venlafaxine: headache, insomnia, dry mouth, agitation, sweating, and urinary retention; blood pressure increase(all events were mild)	
Dimitrios 2012	duloxetine: Nausea 3(15%); somnolence 3(15%); insomnia 1(5%); dizziness 2(10%); dry mouth 2(10%); headache 2(10%)	citalopram: Nausea 4(20%); somnolence 4(20%);dry mouth 2(10%); diarrhea 2(10%);	sertraline: Nausea 5(25%); somnolence 3(15%); insomnia 4(20%); dry mouth 3(15%); diarrhea 4(20%)

Jorge 2004	All adverse events registered during the course of the study were categorized as mild. Transient headaches 6, local discomfort 5, exacerbation of initial insomnia 1. There were no significant differences in the frequency of adverse events between the active and the sham rTMS groups.		
Jorge 2008	active-12k: local pain 1(7%); headache 5(33%); local discomfort 4(27%); anxiety 2(13%); active-18k:local pain 1(3%); headache 7(21%); local discomfort 3(9%);	sham-12k: local pain 1(7%); headache 4(27%); local discomfort 5(33%); sham-18k:headache 3(10%); local discomfort 1(3%);	
Narushima 2010	NR		
Tenev 2010	NR		
Seo 2016	No adverse side effects were reported		
Feng 2004	NR		
Williams 2007	intervention: death 2(ICH); seizure 2; 15 (16%) of intervention group subjects had antidepressant side effects bothersome enough to change medications, and 4 subjects changed medications more than once. The most common of the 39 side effects reported by these 15 subjects was sedation (14), followed by sexual (7), gastrointestinal (6), and anxiety (4) side effects	control: death 1 (myocardial infarction); seizure 1	
Mitchell 2009	NR		

rTMS= Repetitive Transcranial Magnetic Stimulation

TCM= traditional Chinese medicine

Appendix 5: Risk of bias assessments within studies

We used an updated "Risk of bias" tool from the Cochrane Collaboration recommends. This tool addresses seven specific bias domains including methods for generating the random sequence, allocation concealment, blinding of participants and investigators, blinding of outcome assessment, incompleteness of outcome data and selective outcome reporting. Each item is adjudicated within each study and the results are represented in a risk of bias table. We considered allocation concealment adequate if the investigators responsible for patient selection were unable to suspect before allocation which treatment was next. We considered blinding of patients adequate if interventions were described as indistinguishable, or if double-dummy technique was used. We considered blinding of therapists adequate if it was explicitly mentioned in the text that therapists were blinded. We considered incomplete outcome data if it excluded at least one of the randomly assigned patients from the analysis.

Publication bias and selective might affect interventions and comparisons in different ways depending on the clinical context in the network meta-analysis. Using methodology from ecology, attempts have been made to associate the possibility of selection bias with asymmetry measures of the network. Funnel plot asymmetry can be caused by the association between sample size, heterogeneity, and the probability of publication. Sponsorships bias may reflect subtle or less subtle differences in the study designs or the conduct of a trial that only supports the preferred strategy.

(A) Risk of bias graph: review authors' judgements about each risk of bias item presented as percentages across all included studies.

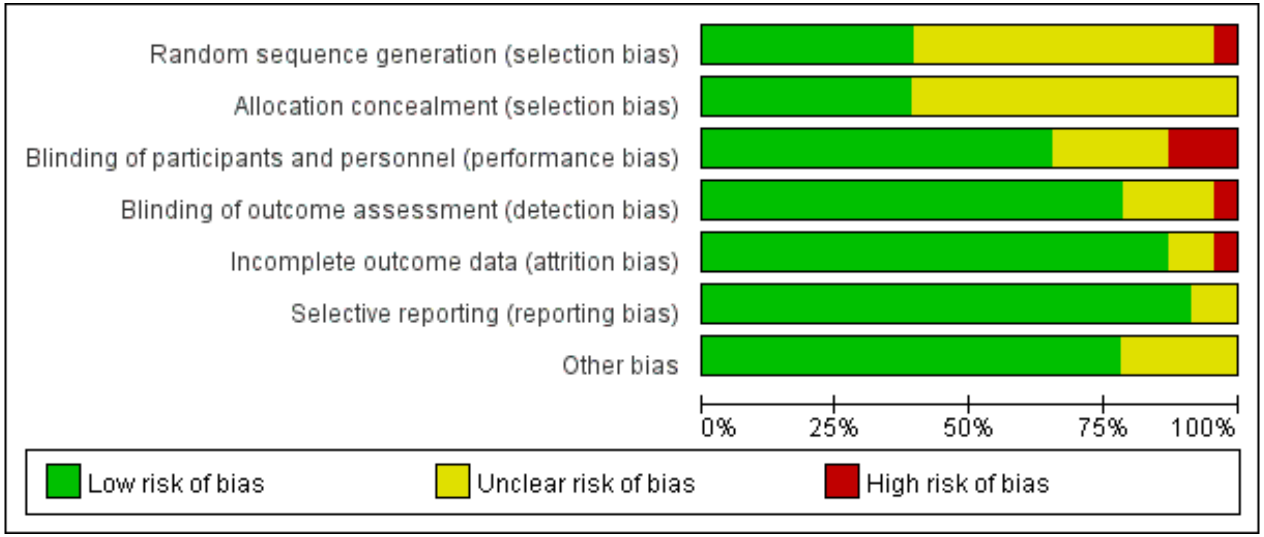

(B)Study-level risk of bias

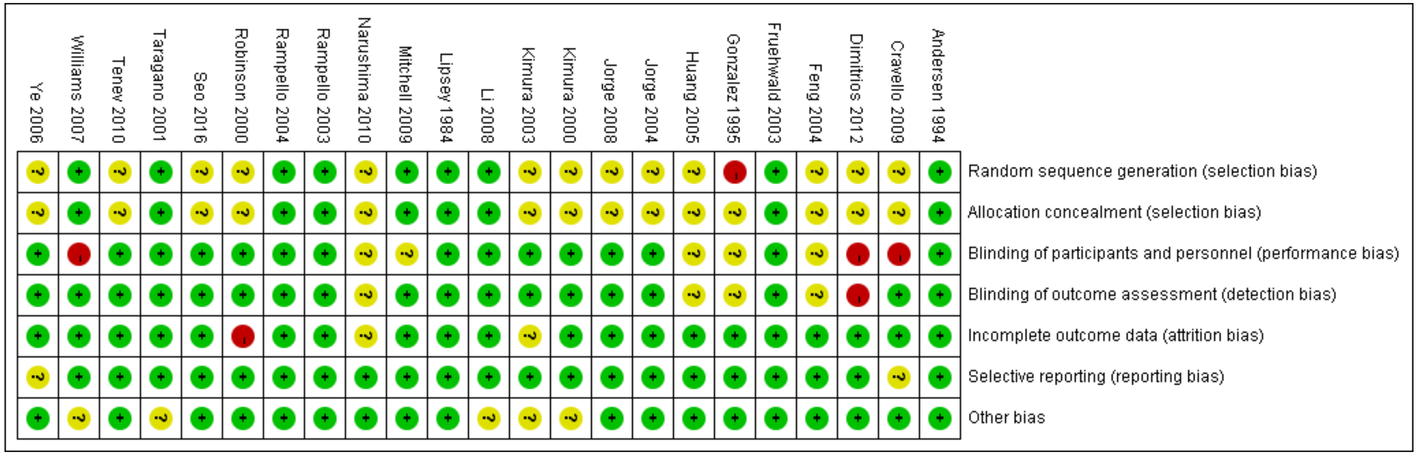

Small-study effects assessed via comparison -adjusted network funnel plots. In this presentation, all studies are centered on the summary effect estimate of their respective comparisons [μXY (logOR for present study)] which is represented by the vertical red line. Individual study-level effect size is represented by yiXY [where X and Y are two study agents]. The green line represents linear regression of the comparison specific differences yi - μXY on the standard error of yi. Outer dotted lines indicate the triangular region within which 95% of studies are expected to lie in the absence of both biases and heterogeneity (logOR ± 1.96*standard error). Please note that this is drawn only for comparisons with 2 or more studies.

(C) Publication bias assessed via funnel plots assessed for reduction of HAMD score between individual treatment

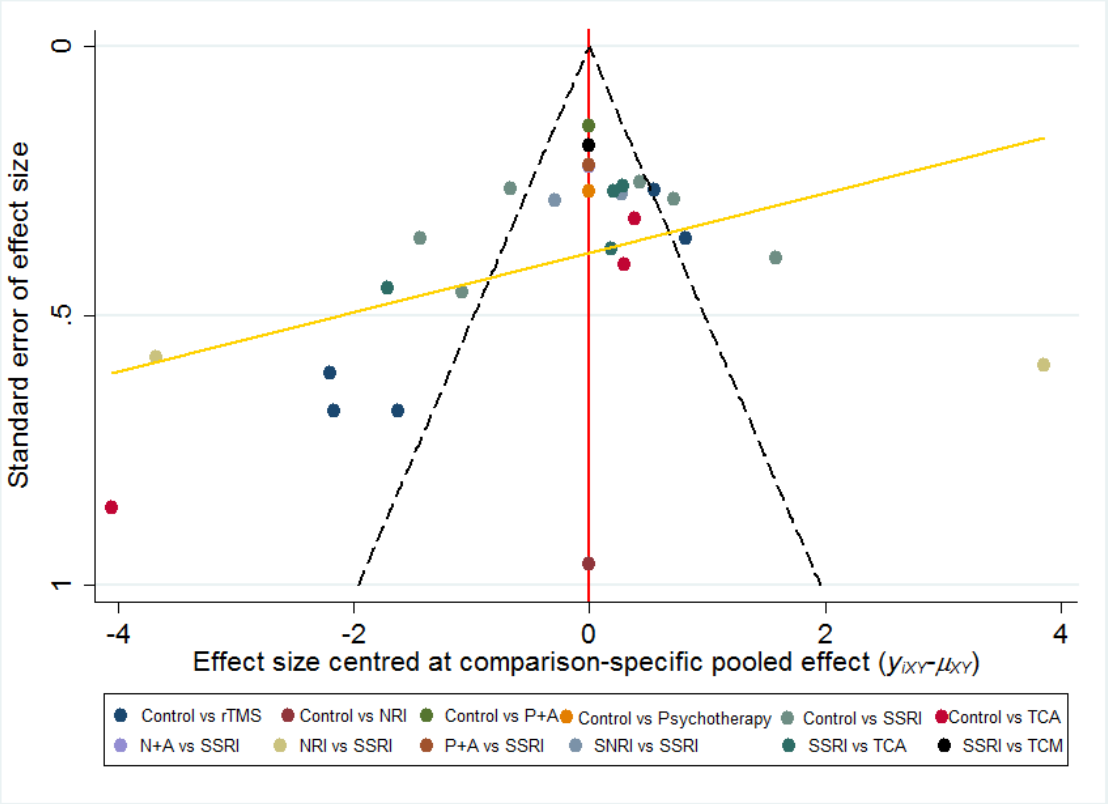

SSRI= selective serotonin reuptake inhibitor. TCA= tricyclic antidepressant. SNRI= serotonin–norepinephrine reuptake inhibitors. NRI= norepinephrine reuptake inhibitor. TCM= traditional Chinese medicine. rTMS= Repetitive Transcranial Magnetic Stimulation. P+A= psychotherapy plus antidepressants. N+A= nimodipine plus antidepressants.

(D) Publication bias assessed via funnel plots assessed for reduction of HAMD score between individual pharmacotherapy

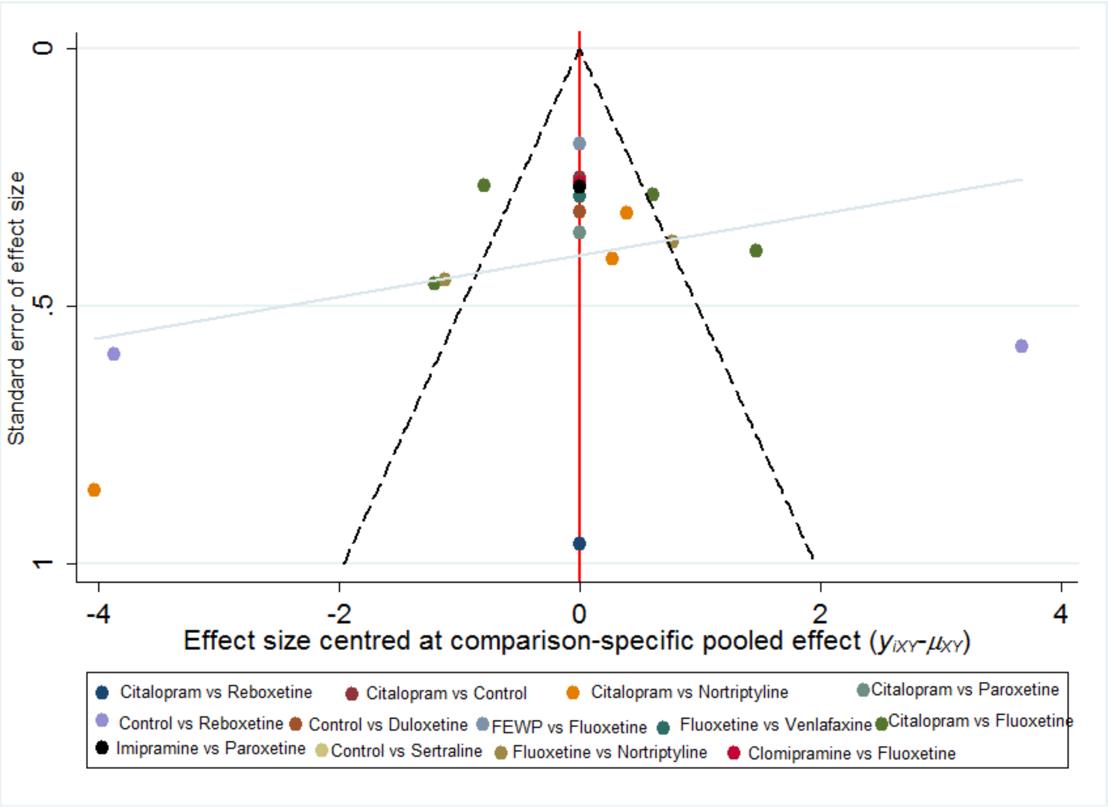

FEWP=Free and Easy Wanderer Plus (a kind of Chinese medicine; its original Chinese Name is Jia-Wei-Xiao-Yao-San)

(E) Publication bias assessed via funnel plots assessed for response rate between individual treatment

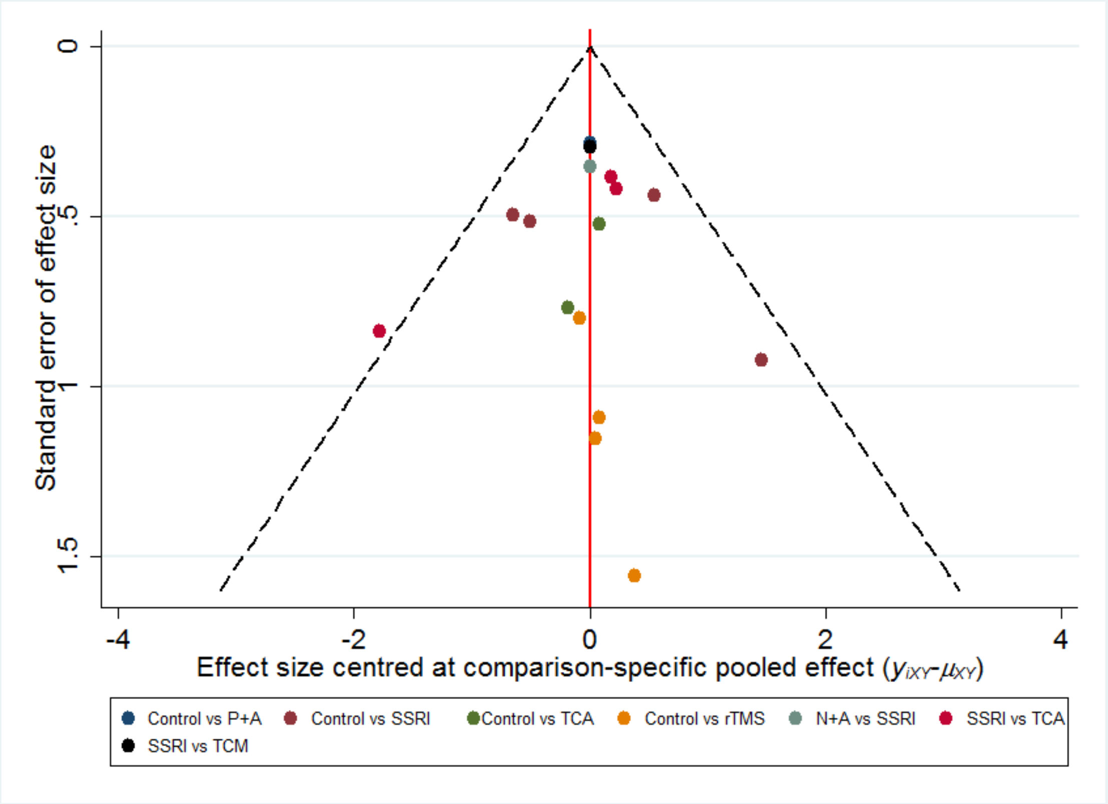

SSRI= selective serotonin reuptake inhibitor. TCA= tricyclic antidepressant. TCM= traditional Chinese medicine. rTMS= Repetitive Transcranial Magnetic Stimulation. P+A= psychotherapy plus antidepressants. N+A= nimodipine plus antidepressants.

(F) Publication bias assessed via funnel plots assessed for remission rate between individual treatment

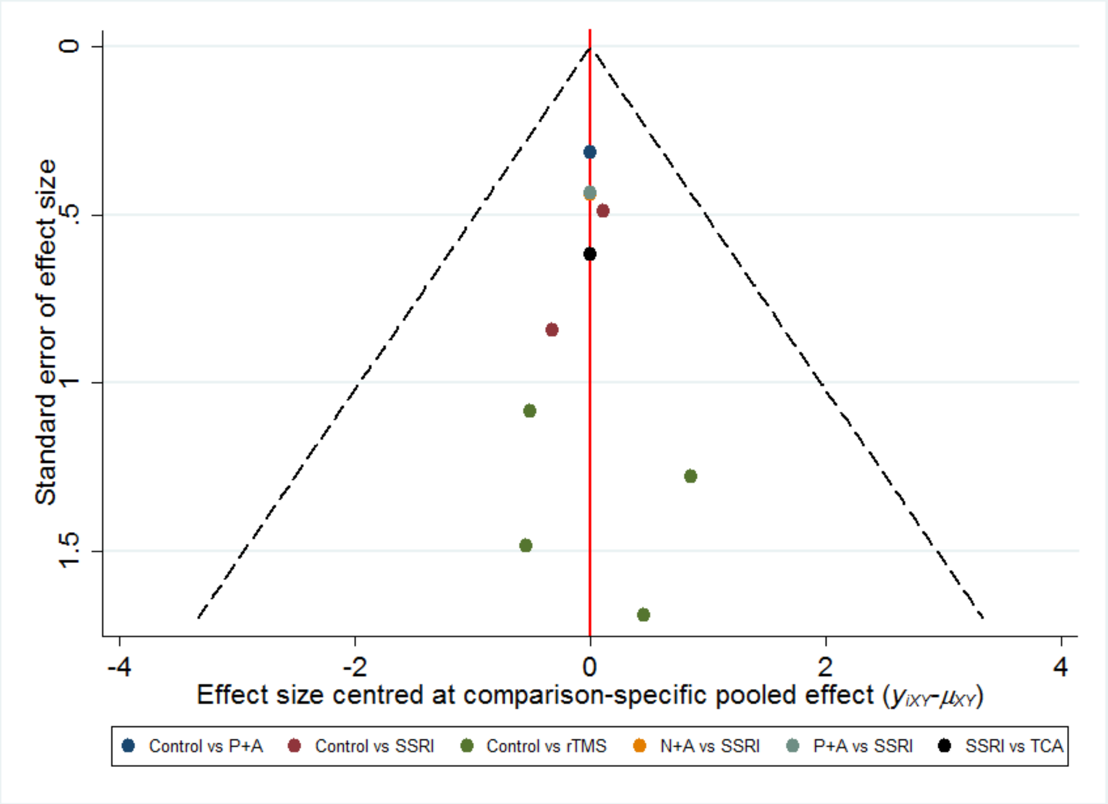

SSRI= selective serotonin reuptake inhibitor. TCA= tricyclic antidepressant. rTMS= Repetitive Transcranial Magnetic Stimulation. P+A= psychotherapy plus antidepressants. N+A= nimodipine plus antidepressants.

Table1. Risk of bias and sponsorship of included studies

Study	Random sequence generation	Allocation concealment	Participant blinding	Investigator binding	Incomplete outcome data	Selective reporting	Other source of bias	Industry sponsorship

Lipsey 1984	Low	Low	Low	Low	Low	Low	Low	Low
Andersen 1994	Low	Low	Low	Low	low	Low	Low	Low
Gonzalez 1995	High[a]	Unclear	Unclear	Unclear	Low	Low	Low	Unclear
Robinson 2000	Unclear	Unclear	Low	Low	High[b]	Low	Low	Unclear
Kimura 2000	Unclear	Unclear	Low	Low	Low	Low	Unclear	Low
Taragano 2001	Low	Low	Low	Low	Low	Low	Unclear	Low
Fruehwald 2003	Low	Low	Low	Low	Low	Low	Low	Unclear
Kimura 2003	Unclear	Unclear	Low	Low	Unclear	Low	Unclear	Low
Rampello 2003	Low	Low	Low	Low	Low	Low	Low	Unclear
Rampello 2004	Low	Low	Low	Low	Low	Low	Low	Unclear

Huang 2005	Unclear	Unclear	Unclear	Unclear	Low	Low	Low	Unclear
Ye 2006	Unclear	Unclear	Low	Low	Low	Unclear	Low	Unclear
Li 2008	Low	Low	Low	Low	Low	Low	Low	Low
Cravello 2009	Unclear	Unclear	High	Low	Low	Unclear	Low	Unclear
Dimitrios 2012	Unclear	Unclear	High	High	Low	Low	Low	Low
Jorge 2004	Unclear	Unclear	Low	Low	Low	Low	Low	Low
Jorge 2008	Unclear	Unclear	Low	Low	Low	Low	Low	Low
Narushima 2010	Unclear	Unclear	Unclear	Unclear	Unlclear	Unclear	Low	Low
Tenev 2010	Unclear	Unclear	Low	Low	Low	Low	Low	Unclear
Seo 2016	Unclear	Unclear	Low	Low	Low	Low	Low	Low

Feng 2004	Unclear	Unclear	Unclear	Unclear	Low	Low	Low	Unclear
Williams 2007	Low	Low	High	Low	Low	Low	Unclear[c]	Low
Mitchell 2009	Low	Low	Unclear	Low	Low	Low	Low	Low

[a]the intervention group and control group were divided randomly, but the 2 intervention group were divided according to their medical condition.

[b]the dropout rate was significantly greater in the fluoxetine group than in the nortriptyline and placebo groups ($\chi2=4.10$, df=1, p=0.04)

[c]52 of the 93 control subjects (56%) took an antidepressant at some time during the 12-week study period

Appendix 6: Network plot for each outcome

(A) Network diagram of eligible comparisons for response rate between individual treatment

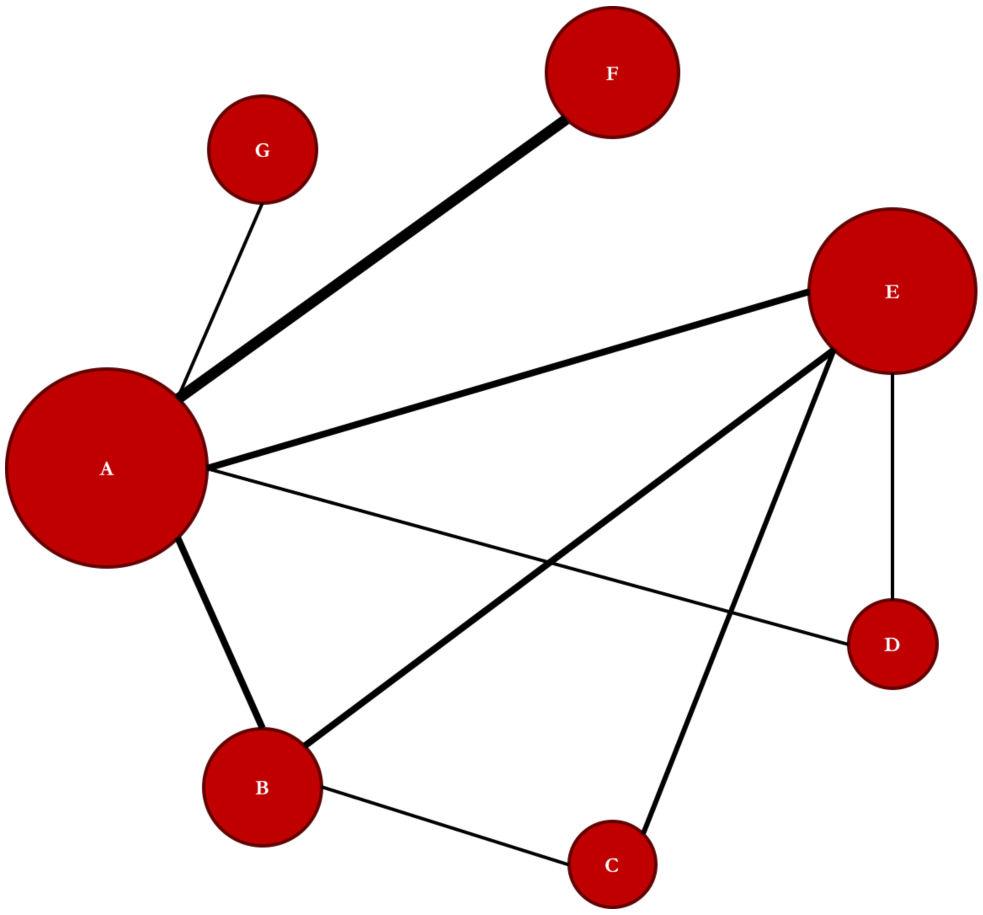

A=control; B=TCA; C=N+A; D=TCM; E=SSRI; F=rTMS; G=P+A

SSRI= selective serotonin reuptake inhibitor

TCA= tricyclic antidepressant

N+A= nimodipine plus antidepressants

TCM= traditional Chinese medicine

rTMS= Repetitive Transcranial Magnetic Stimulation

P+A= psychotherapy plus antidepressants

(B) Network diagram of eligible comparisons for remission rate between individual treatment

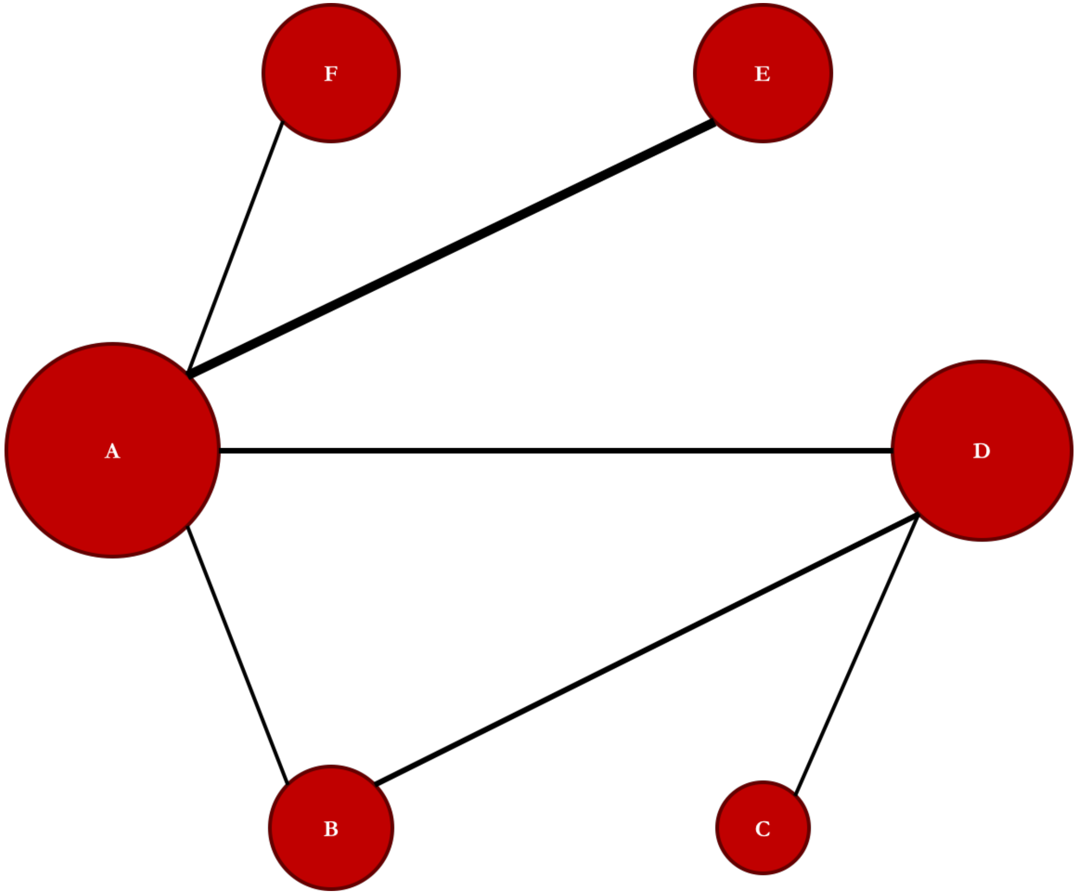

A=control; B=TCA; C=N+A; D=SSRI; E=rTMS; F=P+A

SSRI= selective serotonin reuptake inhibitor

TCA= tricyclic antidepressant

N+A= nimodipine plus antidepressants

rTMS= Repetitive Transcranial Magnetic Stimulation

P+A= psychotherapy plus antidepressants

Appendix 7: Assessment of inconsistency

1. Evaluation of the local inconsistency by loop specific approach

(A) Evaluation of the local inconsistency for HAMD score change

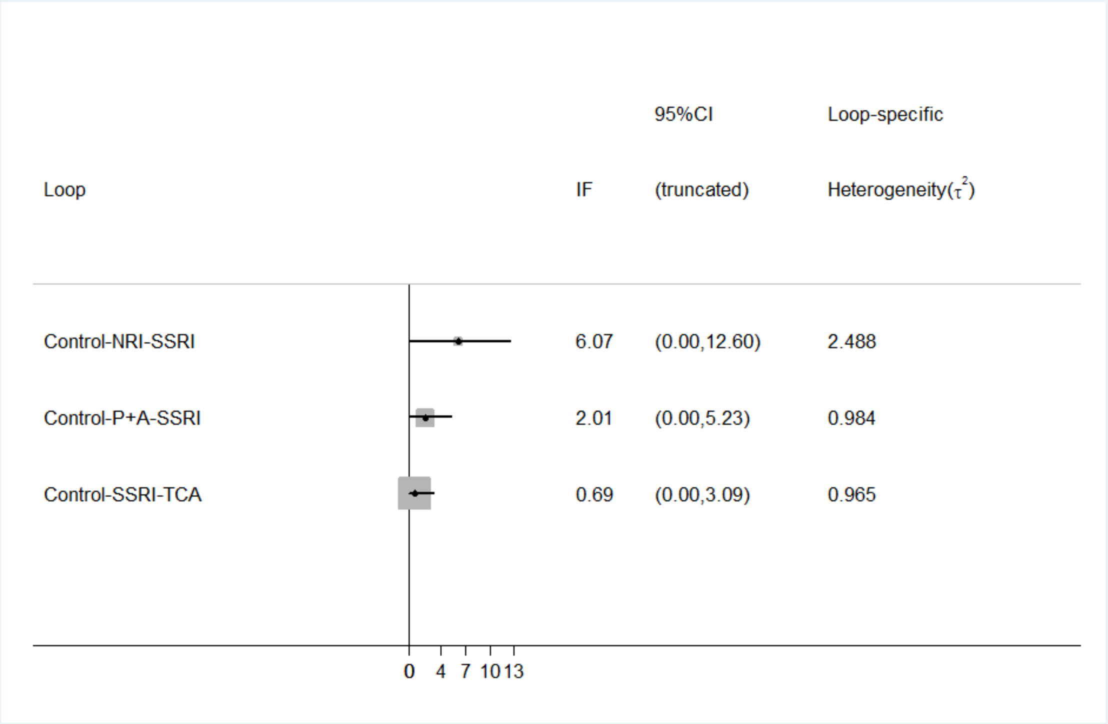

(B) Evaluation of the local inconsistency for patient response rate

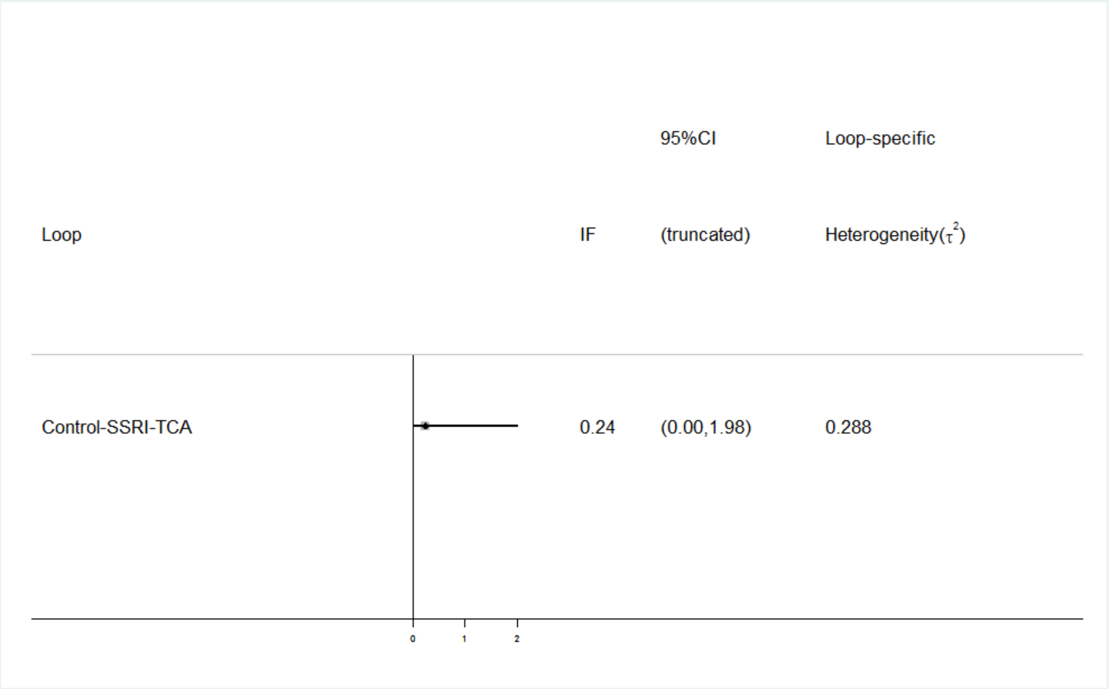

(C) Evaluation of the local inconsistency for patient remission rate

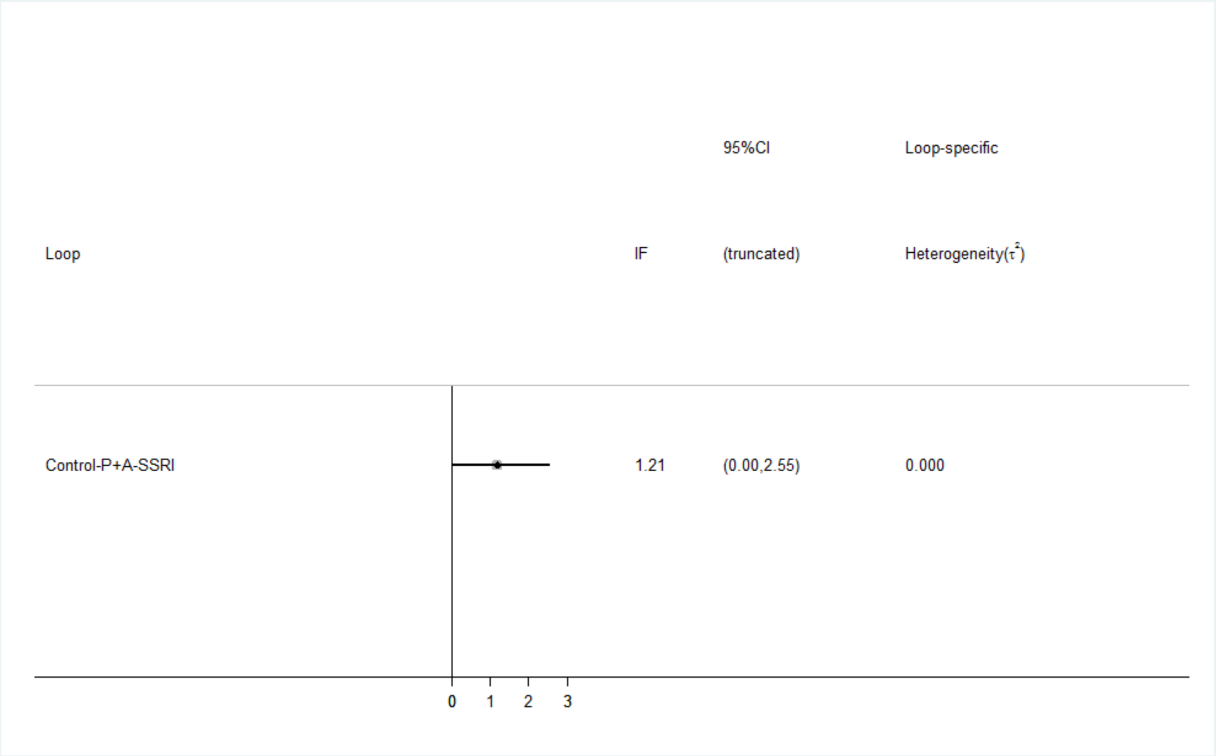

2. **Evaluation of the inconsistency by node-splitting model Tests**

(A) Evaluation of the inconsistency by node-splitting model tests for HAMD score change

**Name**	**Direct Effect**	**Indirect Effect**	**P-Value**
NRI vs. Control	-13.35 (-26.28, -0.22)	-4.76 (-15.01, 5.67)	0.29
NRI vs. SSRIs	0.66 (-8.72, 9.68)	-8.10 (-21.98, 5.79)	0.27

Control vs. P+A	1.98 (-10.90, 14.66)	13.17 (-0.78, 26.73)	0.23
Control vs. SSRIs	6.26 (0.59, 11.99)	7.66 (-1.89, 16.97)	0.79
Control vs. TCA	8.30 (2.82, 13.88)	4.88 (-9.63, 19.84)	0.64
P+A vs. SSRIs	-6.18 (-18.77, 7.04)	4.96 (-8.71, 18.77)	0.23
SSRIs vs. TCA	1.38 (-5.68, 8.48)	-0.71 (-10.67, 9.22)	0.72

(B) Evaluation of the inconsistency by node-splitting model tests for patient response rate

**Name**	**Direct Effect**	**Indirect Effect**	**P-Value**
Control vs. TCA	2.01 (0.80, 3.30)	0.77 (-1.96, 3.43)	0.35
Control vs. SSRI	0.82 (-0.45, 1.93)	2.04 (-0.75, 4.74)	0.35
TCA vs. SSRI	-0.86 (-2.40, 0.56)	-0.95 (-3.14, 1.16)	0.93

(C) Evaluation of the inconsistency by node-splitting model tests for patient remission rate

**Name**	**Direct Effect**	**Indirect Effect**	**P-Value**
Control vs. SSRI	1.43 (-0.25, 3.37)	-0.50 (-3.43, 2.56)	0.16
Control vs. P+A	0.77 (-1.32, 2.80)	2.85 (0.29, 5.72)	0.14
SSRI vs. P+A	1.38 (-0.64, 3.36)	-0.60 (-3.33, 1.90)	0.14

SSRI= selective serotonin reuptake inhibitor

TCA= tricyclic antidepressant

P+A= psychotherapy plus antidepressants

Appendix 8: Treatment ranking and SUCRA plot for each outcome

(A) All treatment

Treatment strategy	HAMD overall change	Patient response rate	Patient remission rate
N+A	0.9487	0.5431	0.6937
NRI	0.6906	-	-
TCA	0.5568	0.8056	0.6733
P+A	0.5242	0.3413	0.3402
SSRI	0.5205	0.4014	0.3841
TCM	0.4592	0.4954	-
SNRI	0.3832	-	-
Psychotherapy	0.3237	-	-
rTMS	0.3041	0.8648	0.8389
Control	0.2910	0.0484	0.0699

Larger SUCRAs denote better procedure.

(B) Pharmacotherapy

Treatment strategy	HAMD overall change	Patient response rate	Patient remission rate
Paroxetine	0.9087	0.7113	0.5221
Imipramine	0.6996	0.6756	0.4728
Reboxetione	0.6868	-	-
Nortriptyline	0.6342	0.8322	-

Duloxetine	0.6105	-	-
Citalopram	0.5992	-	0.9844
Sertraline	0.4332	-	-
Psychotherapy	0.4137	-	-
FEWP	0.4101	0.4659	-
Fluoxetine	0.3280	0.3043	-
Clomipramine	0.3272	0.3221	-
Venlafaxine	0.2421	-	-
Control	0.1511	0.1889	0.0232

Larger SUCRAs denote better procedure.

SSRI= selective serotonin reuptake inhibitor

TCA= tricyclic antidepressant

SNRI= serotonin–norepinephrine reuptake inhibitors

NRI= norepinephrine reuptake inhibitor

N+A= nimodipine plus antidepressants

TCM= traditional Chinese medicine

rTMS= Repetitive Transcranial Magnetic Stimulation

P+A= psychotherapy plus antidepressants

FEWP=Free and Easy Wanderer Plus (a kind of Chinese medicine; its original Chinese Name is Jia-Wei-Xiao-Yao-San)

Appendix 9: Subgroup analysis

(A) Network diagram of eligible comparisons for response rate between individual pharmacotherapy

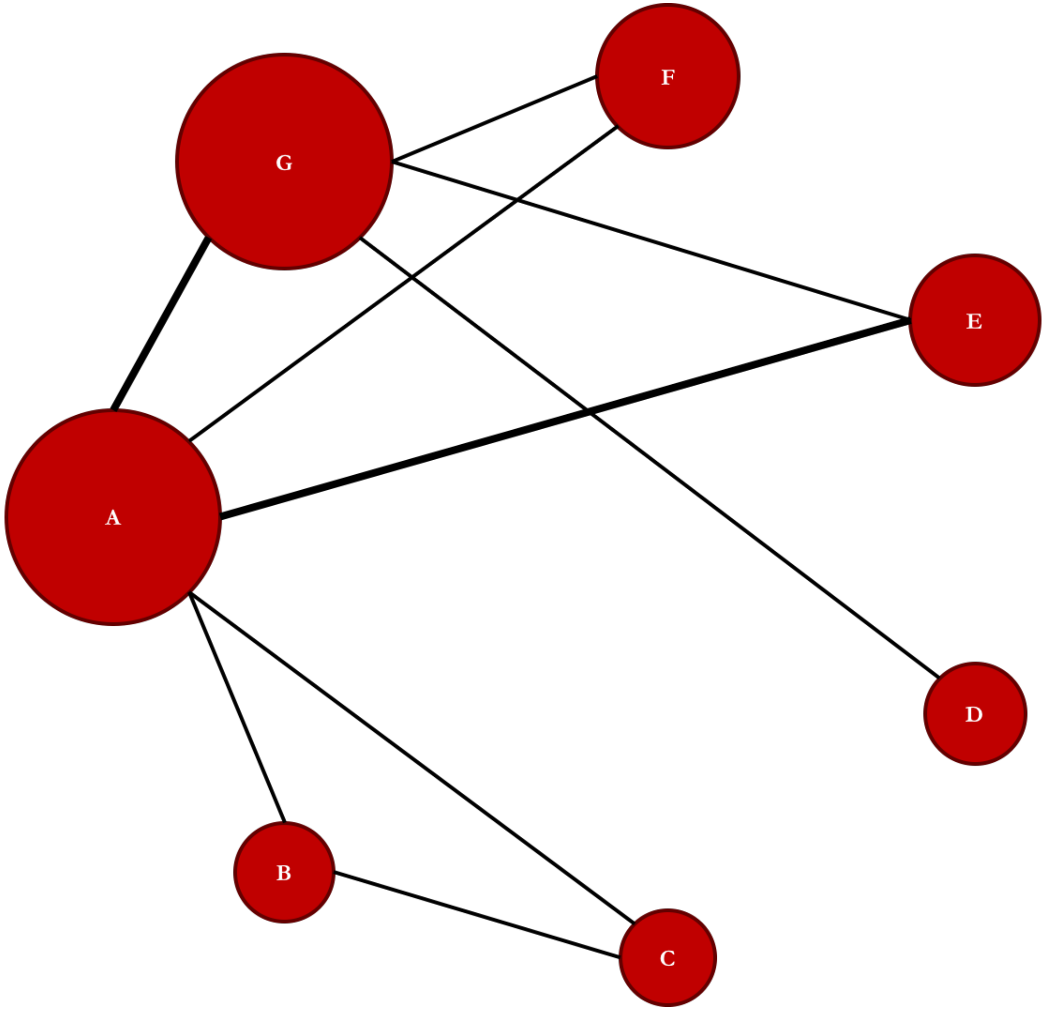

A= control; B=paroxetine; C=imipramine; D=clomipramine; E=nortriptyline; F= Free and Easy Wanderer Plus(a kind of Chinese medicine; its original Chinese Name is Jia-Wei-Xiao-Yao-San) ; G=fluoxetine

(B) Network diagram of eligible comparisons for remission rate between individual pharmacotherapy

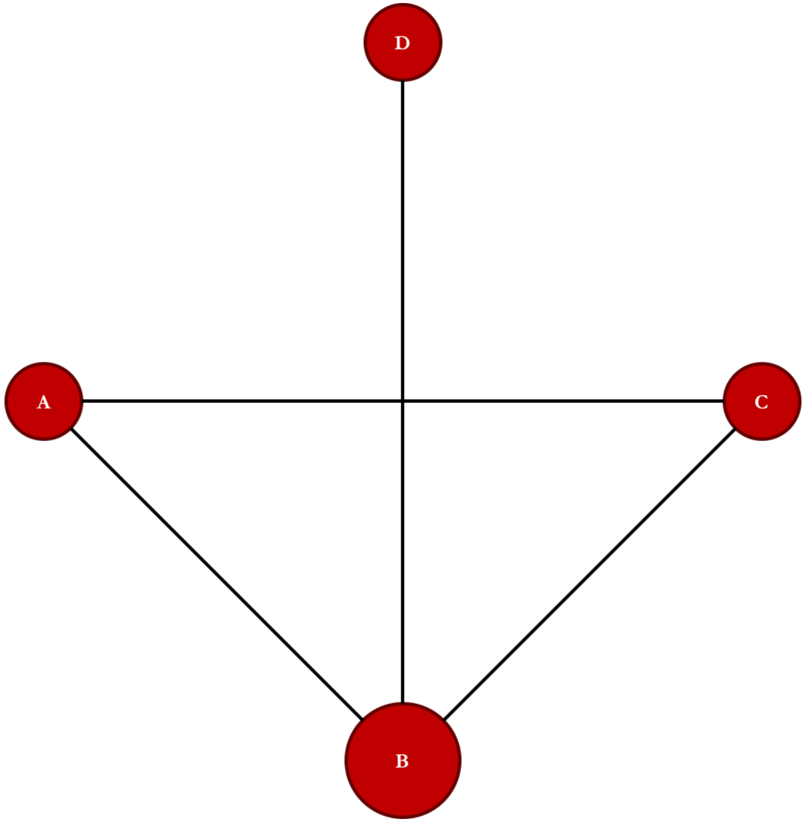

A= control; B=paroxetine; C=imipramine; D=citalopram

Nortriptyline						
1.55 (0.06 - 45.38)	Paroxetine					
1.73 (0.06 - 46.36)	1.16 (0.07 - 18.58)	Imipramine				
4.30 (0.22 - 73.15)	2.99 (0.06 - 100.49)	2.48 (0.05 - 96.71)	FEWP			
7.68 (0.25 - 269.20)	5.00 (0.07 - 444.64)	4.41 (0.07 - 348.19)	1.74 (0.05 - 88.86)	Clomipramine		
7.18 (1.14 - 68.40)	4.95 (0.20 - 131.23)	4.04 (0.18 - 109.08)	1.59 (0.18 - 20.07)	0.94 (0.06 - 17.69)	Fluoxetine	
10.02 (1.82 - 52.56)	6.66 (0.36 - 99.90)	5.73 (0.32 - 86.56)	2.32 (0.21 - 26.02)	1.30 (0.04 - 28.24)	1.43 (0.26 - 5.56)	Control

figure A: Summary odds ratio (OR) and credible intervals from network meta-analysis of response rate of individual pharmacotherapy

Treatments are reported in order of efficacy ranking according to SUCRAs. Comparisons should be read from left to right. The response rate and remission rate estimate is located at the intersection of the column-defining treatment and the row-defining treatment. An OR value below 1 favours the column-defining treatment. To obtain ORs for comparisons in the opposing direction, reciprocals should be taken. Significant results are in bold and underlined. FEWP=Free and Easy Wanderer Plus (a kind of Chinese medicine; its original Chinese name is Jia-Wei-Xiao-Yao-San)

**Citalopram**			
**3.97 (1.29 - 12.04)**	**Paroxetine**		
4.55 (0.82 - 26.81)	1.14 (0.34 - 4.25)	**Imipramine**	
**18.50 (2.75 - 149.70)**	**4.57 (1.00 - 28.00)**	4.04 (0.84 - 23.78)	**Control**

figure B: Summary odds ratio (OR) and credible intervals from network meta-analysis of remission rate of individual pharmacotherapy

Treatments are reported in order of efficacy ranking according to SUCRAs. Comparisons should be read from left to right. The response rate and remission rate estimate is located at the intersection of the column-defining treatment and the row-defining treatment. An OR value below 1 favours the column-defining treatment. To obtain ORs for comparisons in the opposing direction, reciprocals should be taken. Significant results are in bold and underlined. FEWP=Free and Easy Wanderer Plus (a kind of Chinese medicine; its original Chinese name is Jia-Wei-Xiao-Yao-San)

Appendix 10: Post hoc analysis

Study	Location	Participants (N)	Intervention/control (N)	Drop-out rate (%)	Treatment duration	Follow-up	Setting	Center	Depression Diagnostic criteria	Population
Wiart 2000	France	31	Fluoxetine 16	12.5	45 days	45days	inpatient	single center	ICD-10	ITT
			Placebo	0						
Murray 2005	Sweden	123	Sertraline 62	18	6 weeks	26 weeks	inpatient	multi-center	DSM-IV	ITT
			Placebo 61	10						

Study	intervention/control (N)	Mean age (SD)	Gender (%, male)	Mean baseline HAMD/MADRS (SD)	Hemisphere stroke side (%, left)	Depression diagnosis N (%, major depression)	Time since stroke onset
**Wiart 2000**	Fluoxetine(N=16)	66.3(7.1)	56.3	28.5(7.7)	25	100%	47.1(21.6) days
	Placebo(N=15)	68.9(11.6)	40	27.2(6.3)	20		47.7(19.9) days
**Murray 2005**	Sertraline(N=62)	70.7(9.7)	51.6	18.9(6.1)	56.6	66.1	137.3(101.4) days
	Place(N=61)	70.7(10.1)	44.3	19.6(6.1)	33.3	57.4	119.0(92.5) days

Results of Post hoc sensitivity analysis

Post hoc sensitivity analysis of primary main outcome			
Comparisons	Network Meta-Analysis	Rankings of post hoc sensitivity analysis	Rankings of previous analysis
N+A VS CONTROL	9.29(-0.28,18.42)	1	1
NRI VS CONTROL	8.44(2.67,14.26)	2	2
TCA VS CONTROL	7.72(4.13,11.29)	3	3

**P+A VS CONTROL**	**7.41(0.81,14.38)**	4	4
**SSRI VS CONTROL**	**6.60(3.43,9.79)**	5	5
SNRI VS CONTROL	6.22(-2.00,13.70)	6	7
TCM VS CONTROL	5.71(-2.26,13.33)	7	6
Psychotherapy VS CONTROL	4.88(-4.45,14.40)	8	8
rTMS VS CONTROL	3.57(-0.77,7.81)	9	9
Post hoc sensitivity analysis of subgroup analysis			
Comparisons	Network Meta-Analysis	Rankings of post hoc sensitivity analysis	Rankings of previous analysis
**Paroxetine VS Placebo**	**13.26(3.32,23.11)**	1	1
**Imipramine VS Placebo**	**11.30(1.43,21.58)**	2	2
**Reboxetine VS Placebo**	**8.52(1.53,15.24)**	3	3
**Nortriptyline VS Placebo**	**7.75(3.77,12.07)**	4	4
**Citalopram VS Placebo**	**6.63(0.29,12.64)**	5	6
Duloxetine VS Placebo	5.72(-4.64,16.37)	6	5
**Fluoxetine VS Placebo**	**5.15(0.40,9.72)**	7	9
FEWP VS Placebo	4.98(-3.21,13.35)	8	8
Venlafaxine VS Placebo	3.47(-7.20,14.20)	9	11
Clomipramine VS Placebo	3.86(-5.96,14.43)	10	10
Sertraline VS Placebo	3.02(-4.96,11.03)	11	7
